# Recent progress in boron nanomaterials

**DOI:** 10.1080/14686996.2017.1379856

**Published:** 2017-10-16

**Authors:** Takahiro Kondo

**Affiliations:** ^a^ Faculty of Pure and Applied Sciences, University of Tsukuba, Tsukuba, Japan; ^b^ Tsukuba Research Center for Interdisciplinary Materials Science, and Center for Integrated Research in Fundamental Science and Engineering, University of Tsukuba, Tsukuba, Japan; ^c^ Materials Research Center for Element Strategy, Tokyo Institute of Technology, Yokohama, Japan

**Keywords:** Boron, borophene, boron nanotube, boron nanowires, 60 New topics / Others, 105 Low-Dimension (1D/2D) materials

## Abstract

Various types of zero, one, and two-dimensional boron nanomaterials such as nanoclusters, nanowires, nanotubes, nanobelts, nanoribbons, nanosheets, and monolayer crystalline sheets named borophene have been experimentally synthesized and identified in the last 20 years. Owing to their low dimensionality, boron nanomaterials have different bonding configurations from those of three-dimensional bulk boron crystals composed of icosahedra or icosahedral fragments. The resulting intriguing physical and chemical properties of boron nanomaterials are fascinating from the viewpoint of material science. Moreover, the wide variety of boron nanomaterials themselves could be the building blocks for combining with other existing nanomaterials, molecules, atoms, and/or ions to design and create materials with new functionalities and properties. Here, the progress of the boron nanomaterials is reviewed and perspectives and future directions are described.

## Graphical abstract

**Figure:**
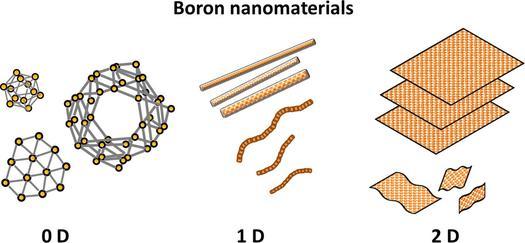

## 1. Introduction

Boron is a promising material in a wide range of fields, such as high-temperature devices and lightweight reinforcing fillers, because of its high hardness, low density, and high melting point (above 2000 °C)[1–3]. Boron is also a unique material with structural complexity owing to its electron-deficient nature, and thus the allotropes of boron are constituted by large-sized unit cells with several atoms in every case. The common feature of three-dimensional (3D) bulk structures of boron is that they are composed of the building blocks of B_12_ icosahedra or icosahedral fragments. The bonding in B_12_ icosahedra is thought to be achieved by the so-called three-center electron-deficient bonds originating from the three valence electrons of boron, where the electron charge is accumulated at the center of a triangle formed by three adjacent boron atoms [2,4,5].

On the other hand, boron is similar to carbon in terms of its capability to form stable covalently bonded molecular networks. Therefore, in parallel with the development of carbon nanomaterials, extensive studies have also been conducted for boron nanomaterials. As early research, in 1966, Olempska et al. [6] reported boron whiskers and needles synthesized by the reduction of boron trichloride with hydrogen at 1300–1800 °C. In 1988–1991, Komatsu et al. [7–12] reported the synthesis of boron films, whiskers, ribbons, and platelets by several experimental methods. Subsequently, boron nanowires, nanotubes, nanoribbons, nanobelts, and/or nanosheets were theoretically predicted in the late 1990s [13–19], followed by the experimental realizations of these boron nanomaterials with detailed analysis of the elements, structures, and/or electronic states from several groups [20]. In the structures of the predicted boron nanomaterials based on the Aufbau principle, the B_12_ icosahedra building blocks are no longer included, owing to their low dimensionality, which results in unusual bonding and intriguing physical and chemical properties [13–45].

One of the recent focal points of boron nanomaterial research is the experimental realization of a single-atomic two-dimensional (2D) layer of boron named borophene by growing the physically evaporated boron atoms on Ag(111) in ultra-high vacuum [46–48]. After the emergence of borophene, several theoretical works about borophene have been reported [49–78], as well as the experimental discovery of the Dirac fermion in borophene on Ag(111) [79].

Specific advantages of the boron nanomaterials, including borophene, for applications such as hydrogen storage, batteries, catalysts, electronics, superconductors, and/or mechanically strong components have been demonstrated, predicted, and/or discussed together with novel structural, electronic, thermal, optical, and mechanical properties [20,49–78,80,81].

In this review, the progress of the boron nanomaterials is described by focusing on experimental reports especially for the synthesis method and its characterization results rather than theoretical predictions. Here, the boron nanomaterials are classified by the following three categories: zero-dimensional (0D), one-dimensional (1D), and 2D boron nanomaterials. The perspective and future directions for boron nanomaterials are then described.

## 2. Zero-dimensional (0D) boron nanomaterials

In the case of carbon, fullerene (C_60_) or higher fullerenes (such as C_70_, C_72_, and C_76_) are often considered as representative 0D nanomaterials, in contrast to the 1D carbon nanotube, 2D graphene, and 3D graphite. However, other small molecules and/or clusters can also be categorized as 0D nanomaterials in terms of the dimensionality in the physical and chemical properties due to their smaller size.

In the case of boron, the Lai-Sheng Wang group systematically elucidated the structures and chemical bonding of size-selected boron clusters produced in gas phase (in vacuum) based on the photoelectron spectroscopy in combination with computational chemistry [24,82–98]. They studied small boron nanoclusters (composed of fewer than 100 B atoms) by comparing small carbon nanoclusters in terms of bonding and structure. For example, all-boron nanotubes [86], all-boron analogues of naphthalene [97], polyenes [90], anthracene [91], phenanthrene [91], and coronene [96] have been observed in their size-selected boron clusters in gas phase and examined in detail to elucidate the structure and bonding by comparing with those of carbon nanoclusters. According to their review [92,98], small atomic boron clusters have planar or quasi-planar structures, stabilized by localized two-center–two-electron (2c–2e) σ bonds on the periphery and delocalized multicenter–two-electron (*n*c–2e) bonds in both σ and π frameworks. Among the observed clusters, a particularly interesting cluster is B_36_, which has been found to possess a planar structure with a central hexagonal vacancy [94]. The hexagonal B_36_ can be viewed as a repeating unit to assemble boron monolayers. These findings provide the first indirect experimental evidence that 2D boron nanomaterials, borophenes, with hexagonal vacancies are potentially viable. Another exciting discovery has been the observation and characterization of the first all-boron fullerenes [95]. Photoelectron spectroscopy revealed that the B_40_^−^ cluster consisted of two isomers with very different electron binding energies. Global minimum searches led to two nearly degenerate isomers competing for the global minimum: a quasi-planar isomer and an unprecedented cage isomer. In the neutral state, the B_40_ cage is overwhelmingly the global minimum, the first all-boron fullerene to be observed, and is named ‘borospherene’ [95,98]. There is evidence that there exists a family of borospherenes with B_28_ being the smallest borospherene [95,98]. It is expected that the pace of discovery will continue to accelerate in boron clusters, and more interesting structures and chemical bonds will be uncovered with heightened research interests and more sophisticated experimental and computational methods [92,98].

In contrast to the great progress in understanding such small boron nanoclusters, the synthesis of these nanoclusters has not yet been established. This is in sharp contrast to the 1D and 2D boron nanomaterials, where production is realized by several methods and superior properties for the applications are shown as described below.

## 3. One-dimensional (1D) boron nanomaterials

### 3.1. Boron nanowires

Nanowires have potential for applications in the electronics field by assembling them as nanoscale electric wires. Thermal and chemical stabilities and uniform conductivity, as well as mechanical strength, are important factors for such applications which are closely related to the morphology and structure of the nanowires. Nanowires are also useful as electron sources by field emission, where the stabilities are again a crucial issue, as well as the tip structure. Boron nanowires were experimentally synthesized earlier among the boron nanomaterials by several experimental methods, which can be classified into three types: chemical vapor deposition (CVD), radio frequency magnetron sputtering, and laser ablation, as summarized in a previous review by Tian et al. [80]. The shape, structure, and bonding nature of synthesized boron nanowires are different depending on the synthesis methods. In any method, the growth of boron nanowires requires a high temperature (500–1500 °C) and the resulting boron nanowires with diameters of 10–300 nm tend to form crystalline structures if the growth is assisted by a catalyst, while amorphous boron nanowires are formed in the absence of a catalyst [80]. In this review, boron nanowires are summarized by classifying amorphous and crystalline boron nanowires with their growth mechanism. The applications of boron nanowires are then briefly introduced.

#### 3.1.1. Synthesis of amorphous boron nanowires

**Figure 1.**
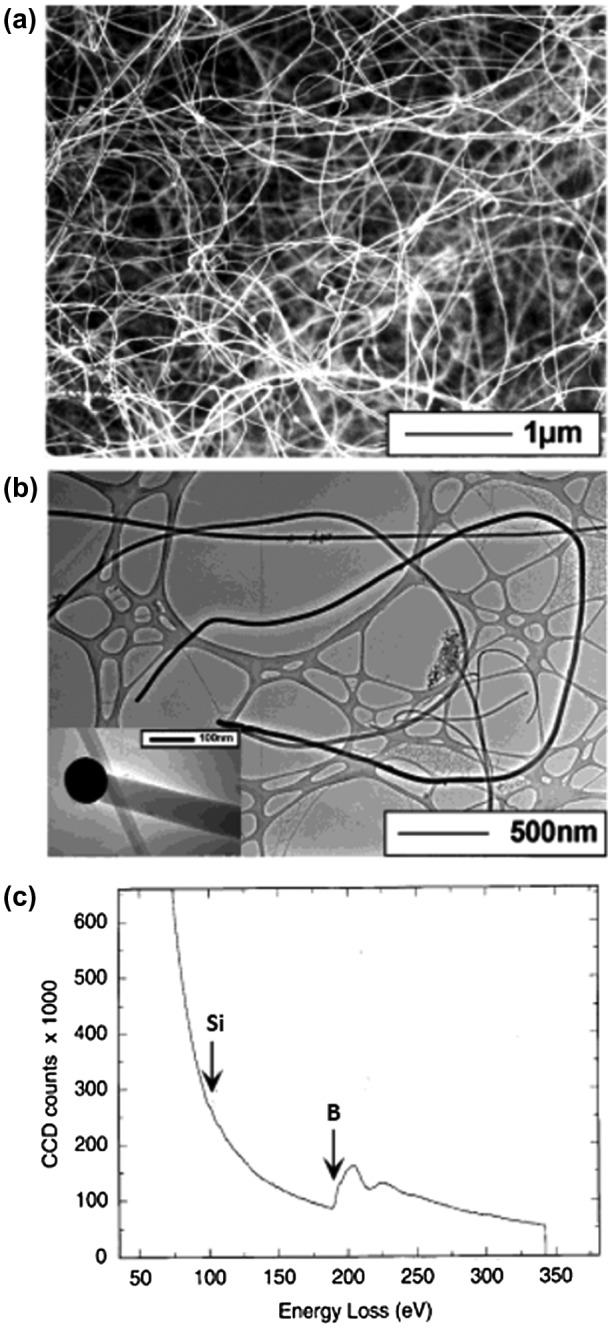
Amorphous boron nanowires synthesized by CVD method. (a) SEM image of the boron nanowires. (b) TEM image of the boron nanowires. The inset shows the tip of one nanowire. (c) EELS spectrum recorded on the boron nanowires. Reproduced from Ref. [99] by permission of John Wiley & Sons Ltd.

Amorphous boron nanowires with several μm length and 50–100 nm diameter were first synthesized and clearly identified by Wu et al. in 2001 [99]. Scanning electron microscopy (SEM) and transmission electron microscopy (TEM) images of their nanowires are shown in Figure 1(a) and (b). The nanowires were obtained as black product by growing on MgO substrate in a sealed quartz tube under BI_3_ vapor at 1000–1100 °C under evacuated condition at 100 mTorr. More specifically, powders of Si, I_2_, and B were put in one end of the tube and a MgO substrate coated by a 5-nm Au thin film was placed in the other end of tube, where a temperature gradient of 100 °C was maintained between source materials and the MgO substrate. At the hot zone, boron reacts with I_2_ and forms BI_3_ vapor. The vapor then decomposed in the low temperature zone to form boron nanowires on MgO. The obtained boron nanowires are amorphous and thus no diffraction spots or rings were observed, even by selected area electron diffraction (SAED). The nanowire tips were composed of Au, B, and Si, while the bodies of the nanowires were composed of B with a small amount of Si, as shown by the electron energy loss spectroscopy (EELS) results (Figure 1(c)). Based on the presence of the droplet of B/Au/Si alloy at the tip, the growth mechanism was suggested as a vapor-liquid-solid (VLS) process with Au as the liquid solvent at high temperature. The authors pointed out that the presence of Si is important to form boron nanowires because nanowires are not formed if Si was removed from the starting materials. The authors used the obtained boron nanowires as an intermediate product for the synthesis of MgB_2_ nanowires [99].

**Figure 2.**
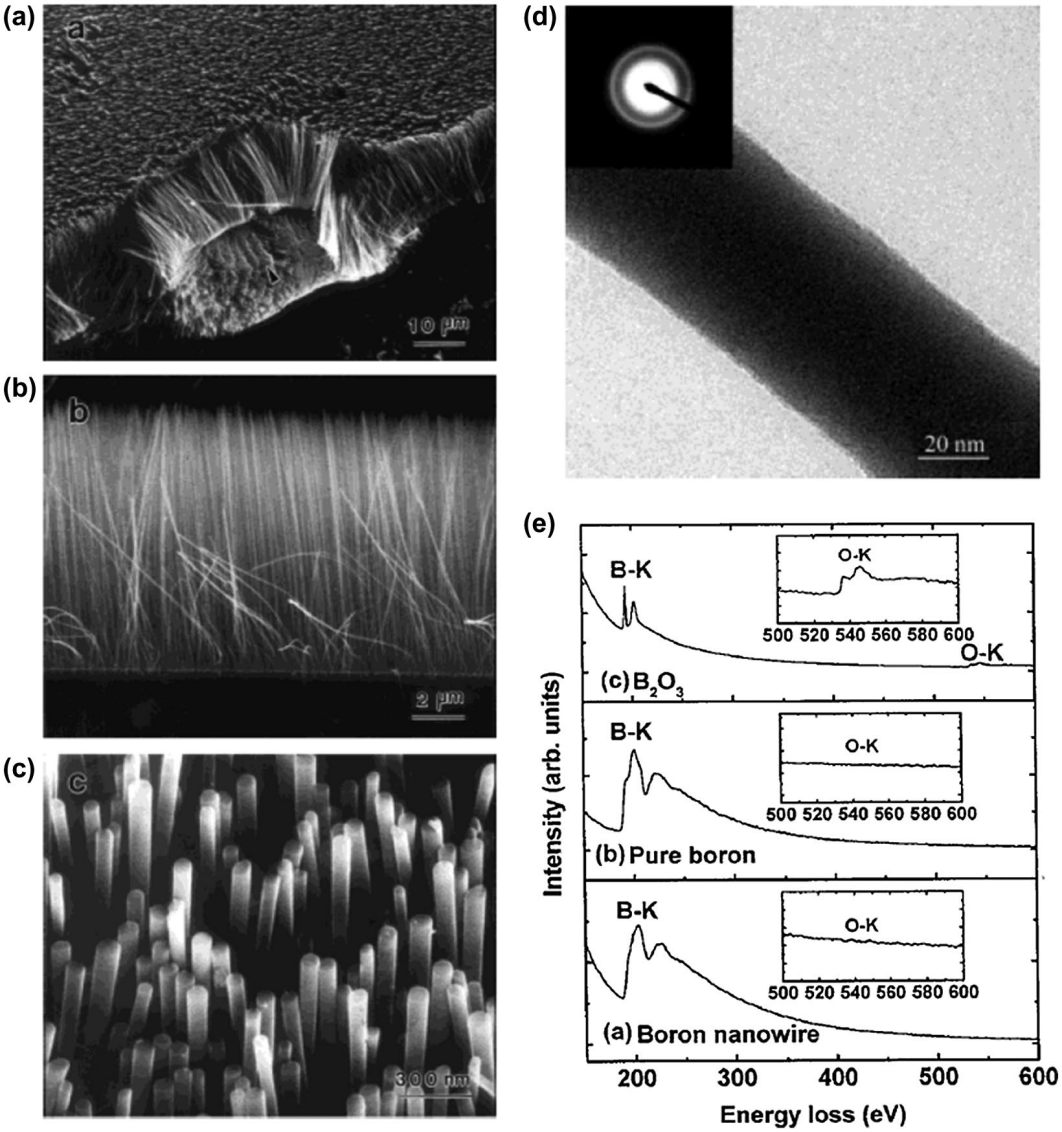
Amorphous boron nanowire arrays synthesized by the radio frequency magnetron sputtering method. (a) Low-magnification SEM image of the aligned boron nanowire arrays grown on Si substrates. (b) Cross-sectional SEM image. (c) High-magnification SEM image. Most of the boron nanowire tips have a platform-shaped morphology with a diameter of 60 ± 80 nm. (d) TEM image of a typical boron nanowire. Inset, SAED pattern taken from the nanowire showing some amorphous halo rings. (e) EELS spectra; a) boron nanowire; b) bulk, pure boron; c) bulk B_2_O_3_. The insets are magnified features of the O-K edges. Reproduced from Ref. [100] by permission of John Wiley & Sons Ltd.

Subsequently, well-aligned amorphous boron nanowire arrays were synthesized by Cao et al. [100] in 2001 in an argon atmosphere by a simple method of radio frequency magnetron sputtering (80 W at 800 °C and 2 Pa for 6 hours), with a target of highly pure boron and a boron oxide mixture (the authors concluded that boron nanowire arrays can also be formed without boron oxide). [Bibr CIT0100]Boron nanowires with diameters of 20–80 nm and lengths of up to several tens of micrometers stand on the substrate Si surface to form well-aligned arrays with self-organized arrangements (Figure 2(a)–(c)). The nanowire arrays can easily be stripped off (Figure 2(a)) with tweezers, suggesting that the boron nanowires are not strongly chemically bonded to the substrate surface. Most of the tips of wires are flat rather than hemispherical in morphology (Figure 2(c)). The TEM and SAED analysis for the boron nanowire peeled off from the arrays show a flat and smooth morphology and amorphous structure (Figure 2(d)). The amorphous phase was further confirmed by high-resolution TEM. The composition of the nanowires was revealed to be boron with a small amount of oxygen (less than 5%) based on EELS analysis (Figure 2(e)). The nanowire arrays in Figure 2 are then concluded to be composed dominantly by boron atoms and no metal elements are included, which is in sharp contrast to the case of nanowires shown in Figure 1.The authors pointed out that a high temperature at the substrate surface (above 700 °C) plays a crucial role in the nucleation and growth of boron nanowires, because the nanowire growth was not observed below 600 °C [100,101]. The growth of boron nanowire arrays is insensitive to the nature of the substrate, because the same size and shape of the boron nanowires were observed after the growth on Si(100), Si(111), SiO_2_, metal plates (Ni, Fe, Co), and metal films (Pd, Au, Ag) on Si. The nucleation and growth of vertically aligned boron nanowires arrays are thus concluded to be entirely self-organized rather than catalyst-assisted processes. Interestingly, merely by increasing the Ar flow rate to 60 cm^3^ min^−1^ (by a factor of approximately two) in the radio frequency magnetron sputtering process, boron nanofeather-like arrays are grown, where each boron wire possesses Y and/or T junctions [102]. The authors concluded that this process might enable the creation of nanometer-size heterojunctions of a wide variety of one-dimensional (1D) nanostructures [103]. Since no crystallization was observed up to a pressure of 103.5 GPa for these boron nanowires, the amorphous structure of boron nanowires is concluded as stable under high pressure at ambient temperature [104]. Here, it should be noted that according to a subsequent report by Wang and Duan [105], the amorphous structure of boron nanowires made by a similar radio frequency magnetron sputtering method can be converted to a rhombohedral crystalline structure (β-boron) with lattice parameters of *a* = 10.95 Å and *c* = 23.82 Å by annealing at 1050 °C under a high vacuum for 3 hours followed by a quench. [Bibr CIT0105]Additionally, Gao et al. [106] reported that crystalline boron nanowires with 10 nm diameter can be directly synthesized by the same method of the radio frequency magnetron sputtering if Au catalysts are present via a VLS growth mechanism even at a lower temperature of 600 °C[Bibr CIT0106]. Concerning the boron nanojunction, later works show that nanojunctions can also be formed by a thermal vapor transfer method [107,108] and an oxide-assisted VLS process [109].

Meng et al. [110] reported the synthesis of amorphous boron nanowires with diameters of 30–60 nm and lengths of several tens of micrometers by a laser ablation method[Bibr CIT0110]. A KrF excimer laser with a wavelength of 248 nm and a frequency of 10 Hz was used to continuously ablate the target of pure boron in an alumina tube at 1300 °C for 5 hours under vacuum with Ar flow (laser energy of 350 mJ per pulse and a pulse duration of 34 ns). Owing to the fact that the target material is only boron powder, the authors concluded that the growth of B nanowires is governed by a vapor-solid (VS) process rather than a VLS process.

**Figure 3.**
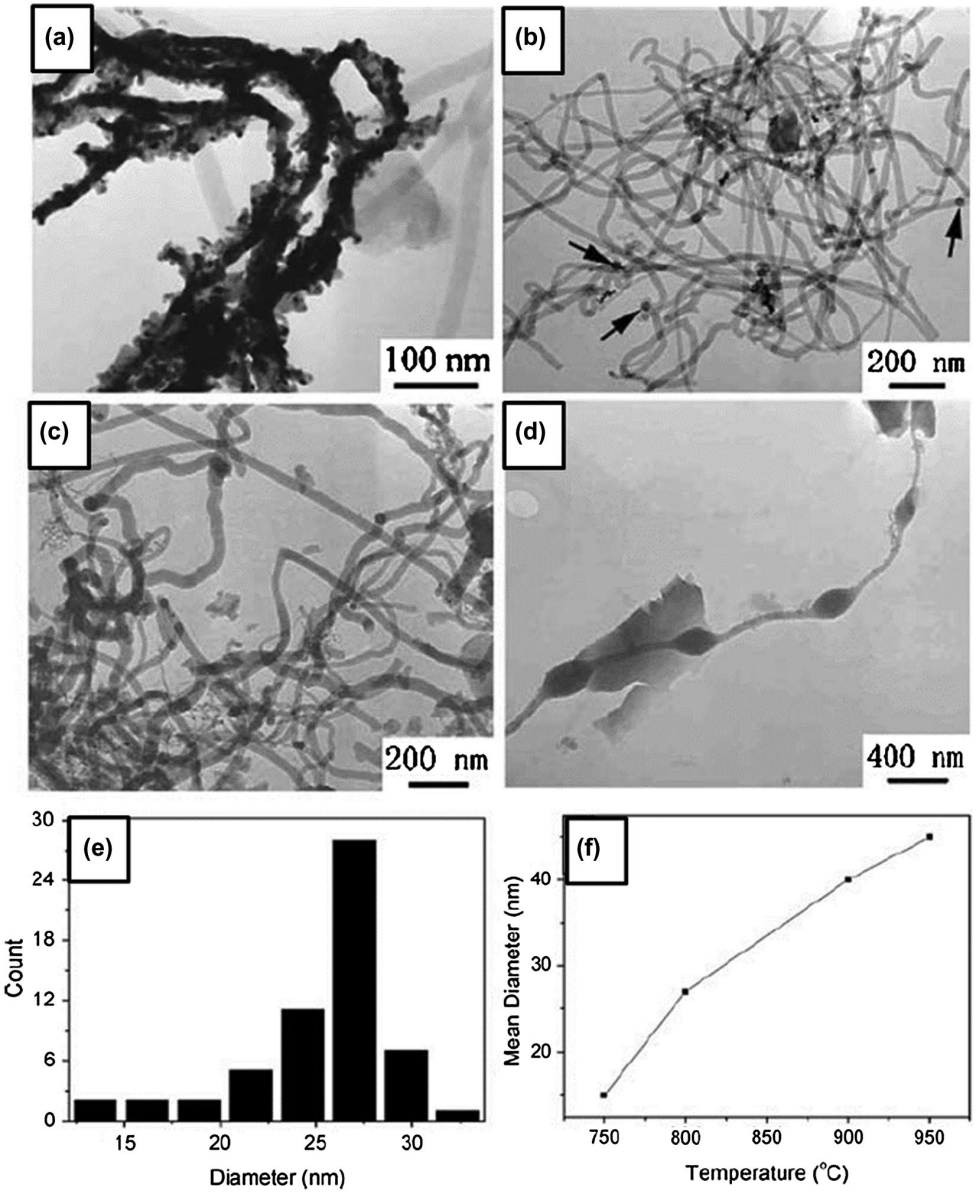
Size control of the diameter of amorphous boron nanowires in CVD method. TEM images of the boron nanowires synthesized at (a) 750 °C, (b) 800 °C, (c) 900 °C, and (d) 950 °C, respectively, while the thickness of the Au film catalyst was 5 nm. (e) A typical diameter histogram for the boron nanowires prepared at 800 °C. (f) Mean diameter of the boron nanowires as a function of temperature. Reproduced from Ref. [111] by permission of SpringerVerlag.

Yang et al. [111] demonstrated control over the morphology and diameter of amorphous boron nanowires, in CVD with VLS growth on silicon substrates, by altering the growth temperature and the thickness of the catalyst Au films on the substrate. A mixture of nitrogen, hydrogen, and diborane with a flow ratio of 10:50:1 was introduced in the quartz tube with Au-coated Si substrate at 750–1000 °C and 5 × 10^4^ Pa for 5 hours. Smooth boron nanowires were fabricated at a temperature ranging from 800 to 900 °C (Figure 3). The diameter of boron nanowires increased slightly from 10 to 50 nm as the growth temperature increased (Figure 3(f)). Boron nanochains with the periodically modulated diameter were also fabricated at 950 °C (Figure 3(d)). As the thickness of the Au films on the Si substrate increases, the diameter and length of the boron nanowires increase dramatically.

#### 3.1.2. Synthesis of crystalline boron nanowires

In parallel with the first reports on amorphous boron nanowires in 2001 [99,100], crystalline boron nanowires were synthesized and identified by Otten et al. (CVD) [112] and Zhang et al. (laser ablation) in 2002 [113].

**Figure 4.**
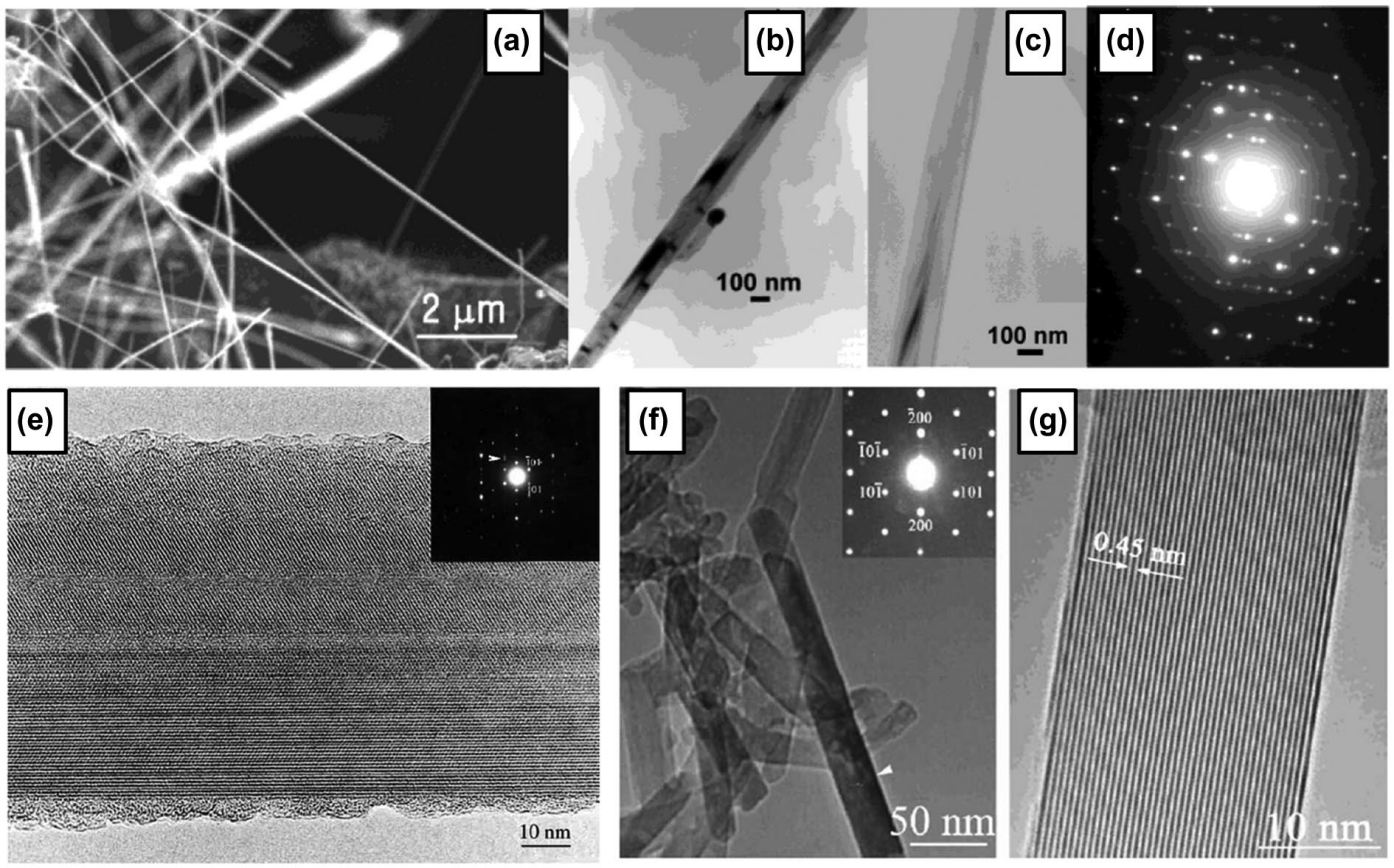
Crystalline boron nanowires. Synthesized by CVD method: (a) SEM image of B nanowires, (b), (c) TEM images, and (d) electron-diffraction pattern of nanowire in (c). Reprinted with permission from Ref [112]. Copyright 2002 American Chemical Society. (e) High-resolution TEM image and its corresponding SAED. Reprinted from Ref. [114]. Copyright 2004 with permission from Elsevier. (f) TEM image of dispersive boron nanowires. The upper right inset is the corresponding SAED pattern of the arrowed boron nanowire. (g) High-resolution TEM image of the arrowed boron nanowire, revealing its single-crystal structure. Reprinted from Ref. [115]. Copyright 2004 with permission from Elsevier.

In the report by Otten et al. (CVD method), a mixture of 5% diborane (B_2_H_6_) in Ar gas was passed at a rate of 15 mL/min for 30 minutes over NiB powder on an alumina substrate in a tube furnace at 1100 °C [112]. The resulting brittle slag has dense entanglements of nanowires (Figure 4(a) with diameters ranging from 20 to 200 nm (mean value ~60 nm) (Figure 4(b) and 4(c)). Based on an electron diffraction pattern (Figure 4(d)), the nanowires were concluded as dense, twinned, whisker crystals with an orthorhombic unit cell of *a* = 9.4 Å, *b* = 7.1 Å, and *c* = 5.4 Å. The conductivity measurements of individual nanowires show semiconducting character with (1.3–5.5) × 10^−5^ (ohm cm)^−1^, which is in the range of 10^−4^–10^−7^(ohm cm)^−1^ reported as that for bulk B.

On the other hand, in the reports by Zhang et al. [113,114], single- and poly-crystalline boron nanowires were obtained as a part of the product of laser ablation[Bibr CIT0113]. A mixture of boron rods with Ni and Co powder was placed as a target in a furnace kept at 1250 °C in a vacuum with Ar flow. A Nd:YAG laser was used for ablation of the target for 30 minutes with 532 nm wavelength, 10 Hz frequency, and 3.5 W power. Nanowires with a diameter less than 100 nm were then observed by SEM and TEM to be grown at the surface of the target. SAED analysis suggests that a portion of the nanowires are single crystal of tetragonal structure with lattice parameters of *a* = 0.875 nm and *c* = 0.506 nm (Figure 4(e)). At one end of the nanowire, a droplet composed of B, Ni, and Co was observed, indicating the VLS mechanism for growing boron nanowires. They concluded that the boron nanowires with diameters ranging from several tens of nanometers to 1 μm can be synthesized by the laser ablation method on the surfaces of the targets, where the synthesis temperatures, intensities of the laser beams, and types of metal catalysts are the main synthesis effect factors on the growth.

Subsequently, well-aligned boron nanowires with single-crystalline structure were reported by Yang et al. in 2003 [115], using nanochannel-Al_2_O_3_ as a substrate and a CVD process. The nanochannel-Al_2_O_3_ was placed in a quartz tube furnace chamber and a mixture of Ar, H_2_, and diborane with a flow rate of 10:10:1 was allowed into the chamber, which was maintained at 1500 Pa and 800 °C. The nanochannel-Al_2_O_3_ substrate with deposited materials was then dissolved in a dilute 1 M NaOH solution at 60 °C. Finally, the solution was dropped onto a copper grid for TEM measurements. Figure 4(f) is a TEM image of dispersed boron nanowires with a diameter of 25 nm. The SAED pattern illustrates that the nanowire is a single crystal tetragonal lattice structure of elemental boron with the lattice constants of *a* = 0.873 nm and *c* = 0.503 nm (inset in Figure 4(f)), which are nearly the same as the lattice parameters of the boron nanowire prepared by laser ablation as described above [113]. The distance between the parallel fringes of boron nanowire observed in high resolution TEM image (Figure 4(g)) is approximately 0.45 nm, corresponding to the spacing of the {1 1 0} planes of B with a 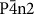 structure. The proposed formation mechanism is as follows: first, diborane gas decomposed into boron atom clusters and H_2_ gas, and boron atoms reacted with the c-Al_2_O_3_ of a nanochannel-Al_2_O_3_ substrate and formed Al_5_BO_9_ layer on the surface of the channels of the nanochannel-Al_2_O_3_, which then prevented boron from reacting with Al_2_O_3_. After that, boron clusters conglomerated to generate boron nanowires by confinement of the channels of the nanochannel-Al_2_O_3_ substrate.

**Figure 5.**
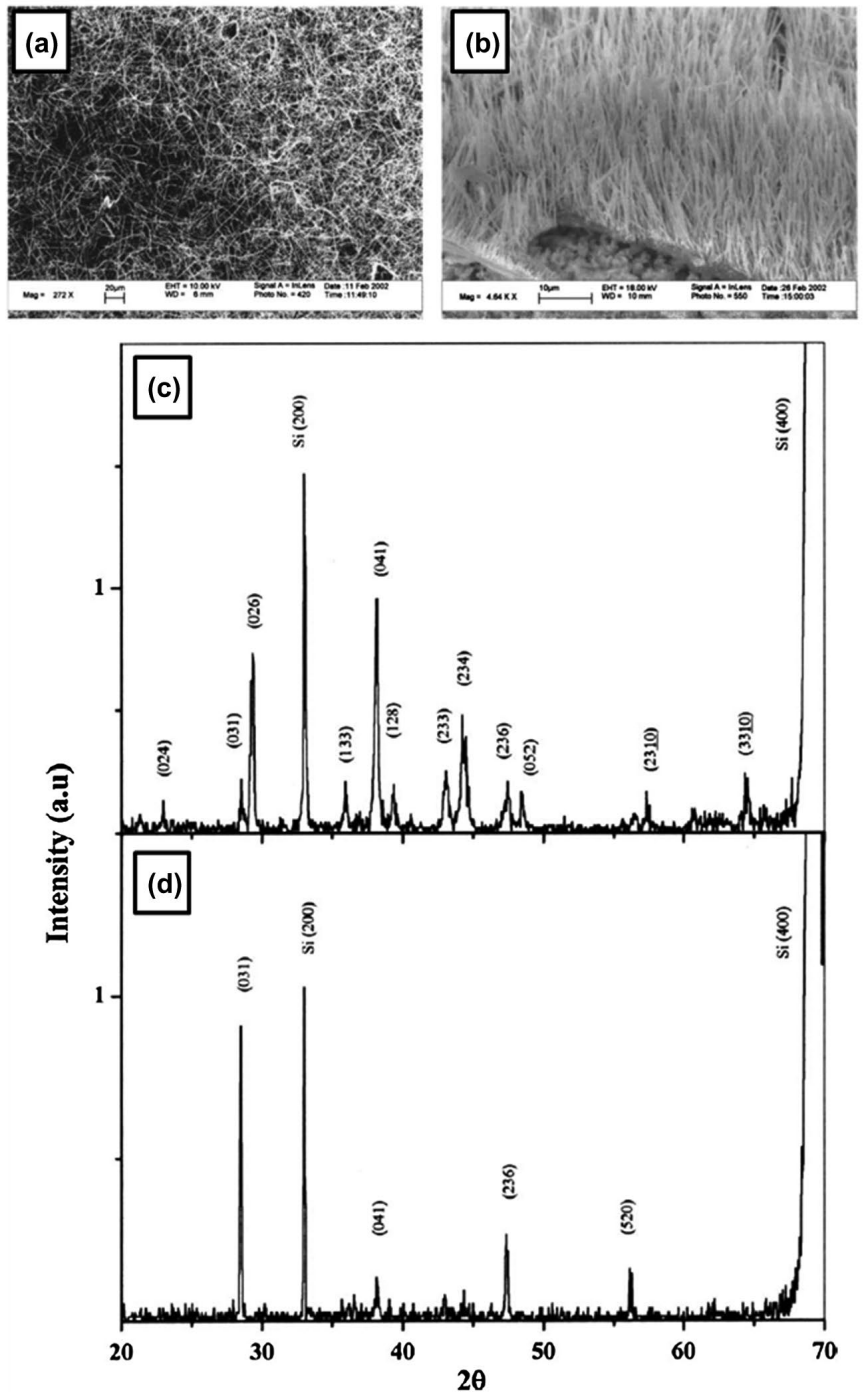
Effect of quenching on crystallinity and alignment of boron nanowires. SEM images (scale bar 10 μm) and XRD patterns of boron nanowires film synthesized at 1100 °C for 30 minutes followed by (a)(c) slow cooling of ~5 °C/min and (b)(d) quench, respectively. Reprinted with permission from Ref. [117]. Copyright 2004 American Institute of Physics.

As described above, the different structure types of the crystalline boron nanowires, i.e. rhombohedral (β-boron) [105,116], orthorhombic [112], and tetragonal [113,114] structures, are formed depending on the synthesis method and conditions. The method to control the growth type of crystal structure of boron nanowires has not yet been well established. However, concerning the quality of the crystalline structure, Yun et al. [117] reported that single-crystalline boron nanowires of a rhombohedral structure (*a* = 10.94 Å and *c* = 23.83 Å) and (031) orientation aligned along the normal of the substrates can be predominantly synthesized by higher-temperature (⩾1100  °C) processing of a thermal vapor transport if it was followed by a quench[Bibr CIT0117]. In the SEM images and X-ray diffraction (XRD) patterns in Figure 5, a sharp contrast can be found between the cases for slow cooling of ~5 °C/min and quench after synthesizing boron nanowires at 1100 °C for 30 minutes, indicating a significant effect of quenching on the improvement of crystallinity and alignment of boron nanowires.

#### 3.1.3. Applications of boron nanowires

**Figure 6.**
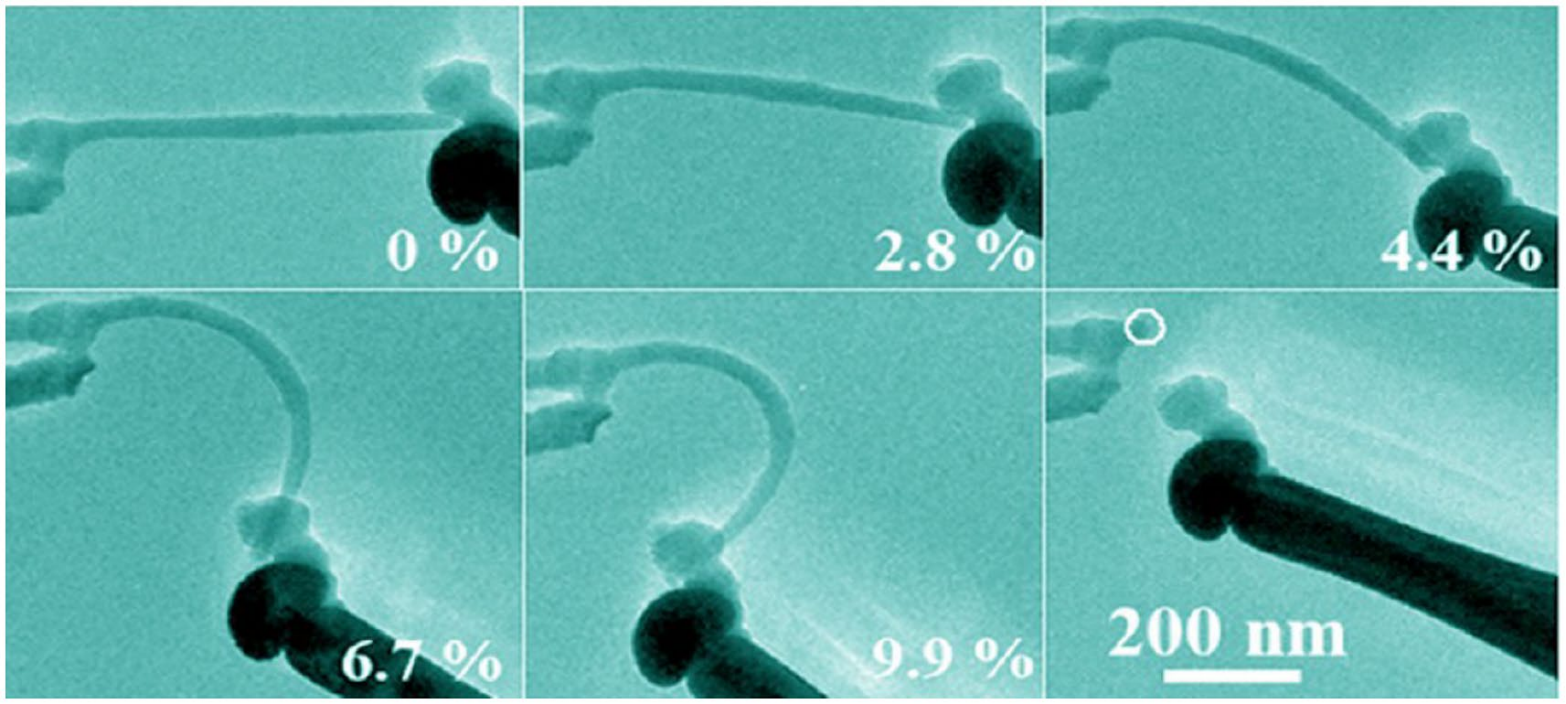
Consecutive TEM images of an individual boron nanowire during different bending stages. Reprinted with permission from Ref. [122]. Copyright 2013 American Chemical Society.

The excellent mechanical properties of individual crystalline boron nanowires have been reported by a few groups [118–122]. According to the report by Liu et al. in 2013 [122], the mean fracture strength and the maximum strain of individual crystalline boron nanowires prepared by CVD method (α-tetragonal lattice, [001] growth direction) were measured to be 10.4 GPa and 4.1%, respectively, during the tensile tests[Bibr CIT0122]. The average Young’s modulus was calculated to be 308.2 GPa under tensile and compression tests. Bending experiments for individual boron nanowires revealed that their maximum bending strain could reach 9.9% (Figure 6) and their ultimate bending stress occurred at 36.2 GPa. These values are much higher than those of Si and ZnO nanowires, which are known for their high bending strength. The boron nanowires also show a very high specific fracture strength of 3.9 GPa·cm^3^/g and specific elastic modulus of 130.6 GPa·cm^3^/g, which are one to two orders of magnitude larger compared to many reported nanostructures [122]. These results suggest that the boron nanowires are a promising material for application as lightweight reinforcing fillers.

**Figure 7.**
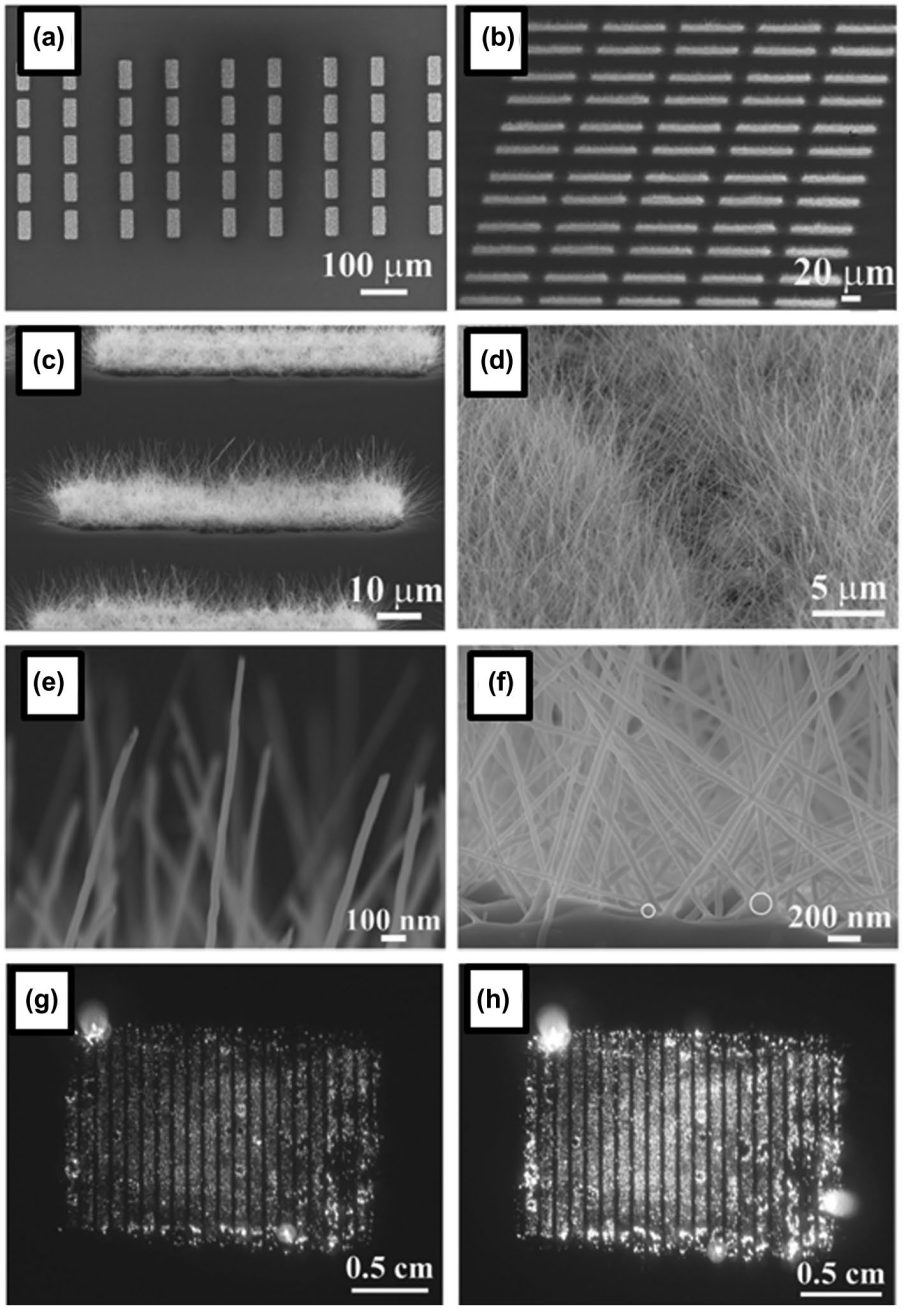
(a) SEM image of crystalline boron nanowire patterns. (b) Side view SEM image of uniform boron nanowire patterns. (c), (d) Side view SEM image of boron nanowires at the edge and at the inner portion of the pattern. (e), (f) SEM images of the tip and the end of boron nanowires. The white circles indicate the catalysts’ sites. (g), (h) Field emission images of patterned boron nanowires at a current density of 1.4 mA/cm^2^ and 2.1 mA/cm^2^. Reproduced from Ref. [129] by permission of John Wiley & Sons Ltd.

The excellent field emission properties of boron nanowires, such as low turn-on field and high current endurance, have been reported [123–129]. For practical applications in field emission displays, there are two important additional issues: (i) patterned growth of boron nanowire arrays over a large area; and (ii) uniformity of physical properties of grown boron nanowires. According to the report by Liu et al. in 2014 [129], uniform crystalline boron nanowires (α-tetragonal lattice and the growth direction along the [001] orientation) with square (25 μm × 60 μm) patterns have been grown over a large area by CVD on a Si substrate with Ni catalysts patterned by an ultraviolet lithography technique (Figure 7(a) and (b)) and the patterns show bright and uniform performance of field emission[Bibr CIT0129]. The side view in Figure 7(c) shows the high and uniform density of boron nanowires in each square of their prepared pattern. Most of the boron nanowires have an average length of approximately 6 μm with a uniform diameter of 30–40 nm (Figure 7(c) and (d)). There are no catalysts at the tips of the boron nanowires (Figure 7(e)), while Ni catalysts exist between the ends of the boron nanowires and the substrate (shown as white circles in Figure 7(f)), indicating that the boron nanowires are formed by a base-up growth mode. The authors also measured *I*–*V* curves of individual boron nanowires and estimated the electronic conductivity as 1.66 × 10^−2^ (ohm cm)^−1^ which is a few orders of magnitude greater than that of bulk B of 10^−4^–10^−7^(ohm cm)^−1^. For the patterned boron nanowires, a turn-on field of 4.3 V/μm (at 10 μA/cm^2^ emission current density) and a threshold field of 10.4 V/μm (at 1 mA/cm^2^ emission current density) have been reported. These values are comparable to those of many excellent cathode nanomaterials, such as ZnO nanowires, WO_3_ nanowires, and AlN nanowires, but are inferior to those of carbon nanotubes, graphene, and LaB_6_. Figures 7(g) and 7(h) show field emission images of patterned boron nanowires at a current density of 1.4 mA/cm^2^ and 2.1 mA/cm^2^. Nearly all patterns are simultaneously involved in the emission process and the emission brightness increases with the emission current density. It is worth noting that both the distribution of the emission patterns and their brightness distribution are very uniform owing to the uniform growth of boron nanowires.

### 3.2. Boron nanotubes

**Figure 8.**
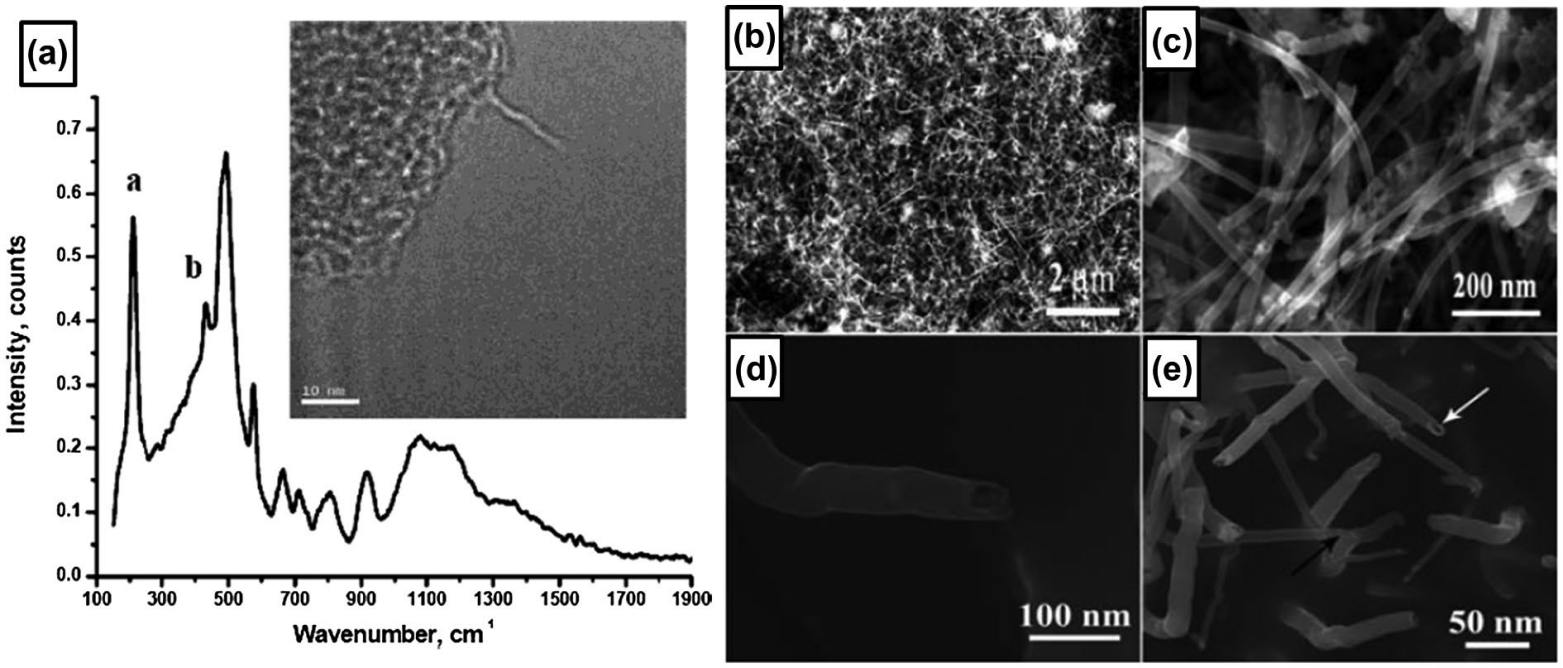
(a) Raman spectrum and TEM image (inset) of boron nanotube. Owing to the extreme beam sensitivity and charging of the sample, the image is blurred and out of focus. Reprinted with permission from Ref. [130]. Copyright 2004 American Chemical Society. (b) Low-magnification SEM image of large-area boron nanostructures. (c) Magnified SEM image of the boron nanostructures. (d) SEM image of a boron nanotube tip. (e) High-resolution SEM image of the boron nanowires and boron nanotubes at the growth stage. Black and white arrows indicate a boron nanotube and a boron nanowire, respectively. Reproduced from Ref. [131] by permission of The Royal Society of Chemistry.

After several theoretical studies in 1990s predicted the structure and intriguing properties of boron nanotubes, such as metallic conductivity exceeding that of carbon nanotubes [10–16], the CVD synthesis of a pure boron single-wall nanotube was reported by Ciuparu et al. in 2004 [130]. They used a magnesium-substituted mesoporous silica template (Mg–MCM-41) as a catalyst and a mixture of BCl_3_ and H_2_ gases (volumetric ratio of approximately 1:6) as a source. The gas mixture was introduced at a rate of 1.5 L/min for 45 minutes in a quartz reactor (inner diameter 6 mm) with the Mg–MCM-41 catalyst at a steady state temperature of 870 °C under a continuous flow of hydrogen. The reactor was then cooled to room temperature under He flow. Figure 8(a) (inset) shows a TEM image of the single-wall boron nanotube obtained on the catalyst, which shows a tubular structure. The Raman spectrum shown in Figure 8(a) contains distinct peak at 210 cm^−1^, which was attributed to the characteristic radial breathing mode of the tubular structure. The author also attributed the spectral features between 300 and 500 cm^−1^ (peak b) to tubular structures. Based on EELS results, the structure is revealed to be composed of pure boron. Thus, the observed structure is concluded as a pure boron single-wall nanotube. For the synthesis method, the authors pointed out the importance of the presence of Mg in the catalyst, since no boron nanotube formation was observed when they used a pure siliceous MCM-41.

The fabrication of a larger quantity of crystalline boron nanotubes was reported by Liu et al. in 2010 [131]. They synthesized boron nanotubes with diameters ranging from 10 to 40 nm and an average length of several micrometers by growing on Fe_3_O_4_ nanoparticles with diameters less than 10 nm through high-temperature solution phase reaction at 1000–1200 °C for 4 hours, where a mixture of B_2_O_3_ and boron powders with mass ratio of 4:3 was used as the source material. Figure 8(b) shows the densely packed boron nanostructures on the silicon substrate, with lengths in the range of 2–4 μm. A typical high-resolution SEM image of the boron nanotubes is shown in Figure 8(c), in which some boron nanowires are mixed in the product. A side view of a typical boron nanotube is given in Figure 8(d), showing that the free end of the boron nanotube is open. A SEM image of the synthesized product with a growth time of 10 minutes is shown in Figure 8(e). The white and black arrows indicate the short boron nanotubes and boron nanowires, respectively, which coexisted at the early stage of growth. Initial growth precursors of the boron nanotubes are ascribed to the alloy nanodroplets of boron-magnetite and boron nanostructures are believed to be grown by the root-growth VLS mechanism. Based on the SAED pattern, the boron nanowires are revealed to be composed of perfect single crystals with an α-tetragonal structure and their growth direction is along [001]. The as-synthesized boron nanotubes are multi-layered nanotubes with the spacing between two adjacent layers of approximately 3.2 Å. The measurements of individual boron nanotubes show that the conductivity is in the order of 10^2^ (ohm cm)^−1^, showing the metallic transport property of boron nanotubes. The conductivity is four orders of magnitude larger than that of the largest conductivity of boron nanowires described above. The individual boron nanotube also shows excellent field emission properties, with a high stable current of approximately 80 μA and a current density of 2 × 10^11^A/m^2^ which are very close to those of carbon nanotubes.

### 3.3. Boron nanobelts and nanoribbons

Nanobelts and nanoribbons are materials that are macroscopically categorized as 1D rather than 2D (nanosheets), since they have a rectangular cross-sectional shape and have a width-to-thickness ratio greater than one, which is also different from simple 1D tubes or wires. Nanoribbons have significantly larger width-to-thickness ratios (10–100) compared with nanobelts (~5), and thus they should have different chemical and physical properties, depending on their size and shape as well as their bonding nature.

#### 3.3.1. Boron nanobelts

**Figure 9.**
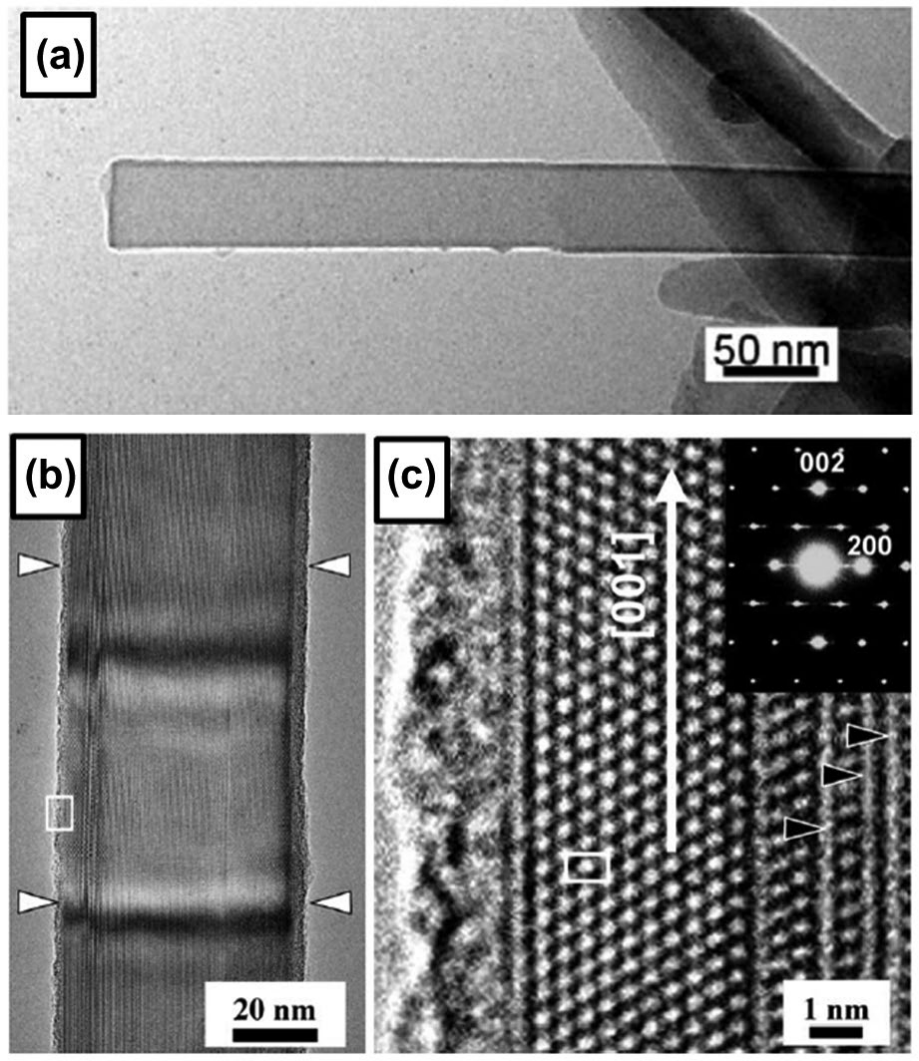
(a) TEM image of a boron nanobelt tip. (b) TEM image of a 55 nm-wide boron nanobelt. The surface of this nanobelt is sheathed with amorphous phase, as indicated by white arrows. (c) TEM image of white rectangular region in (b). Some stacking faults, indicated by black arrows, can be seen along the growth direction. A detailed analysis of the corresponding electron diffraction pattern shown in the inset indicated that the crystal structure is tetragonal and the growth direction of this nanobelt is the [001] direction. Reprinted from Ref. [132]. Copyright 2003 with permission from Elsevier.

The synthesis of single-crystalline tetragonal boron nanobelts was first reported by Wang et al. in 2003 [132]. They synthesized boron nanobelts by a laser ablation method (Nd:YAG laser with 355 nm wavelength, pulse width of 5–7 ns and 10 Hz pulse frequency) in a furnace without using a catalyst or boron vapor. The laser was introduced through a quartz glass window to the target of a hot-pressed boron pellet on a rotating holder placed at the center of the furnace. The boron nanostructures were deposited on the quartz glass substrate at 800 °C under Ar flow at 25 Pa. A TEM image of a typical obtained boron nanobelt (Figure 9(a)) shows flat edges without the presence of nanoparticles and inhomogeneity, where EELS confirm that the nanobelts are composed of boron. Boron nanobelts have a width-to-thickness ratio of approximately 5 (several tens of nanometers to approximately 150 nm) and several micrometers to the order of millimeters in length. Figures 9(b) and 9(c) show the boron nanobelt with tetragonal crystalline structure (*a* = 0.884 nm and *c* = 0.500 nm, [001] growth direction) with 55 nm width, where an amorphous structure can be seen along the surface of the nanobelt with 2–4 nm thickness. Some stacking faults were also observed along the growth direction, as shown by black arrows in Figure 9(c). The authors explained that the growth was progressed by an oxide-assisted-growth (OAG) mechanism, in which an oxide layer is presumed to be molten or near-molten, which might enhance atomic absorption, diffusion, and desorption. The synthesized crystalline boron nanobelts were revealed to be p-type semiconductors with electrical conductivity of the order of 10^−3^ (ohm cm)^−1^ at room temperature, which can be enhanced by a factor of 100–500 by doping Mg [133–135].

In 2008, α-tetragonal crystalline boron nanobelts with [001] growth axis were also reported to be synthesized by combining the methods of e-beam evaporation and plasma ion bombardment [136]. Here, the boron was evaporated on an oxidized silica substrate coated with Au, which was heated to 1100 °C followed by bombardment via a helicon plasma source. Most of the resulting nanobelts had a width of 0.1–1 μm and a thickness of 50–100 nm, while a few nanometer-sized belts, such as those with a width of 30–100 nm, thickness of 10–15 nm, and length of a few to over 15 μm were also formed.

On the other hand, in 2008, rhombohedral crystalline boron nanobelts with a [111] growth direction and amorphous boron nanobelts were synthesized separately by controlling the condition of the method with a vapor-liquid-solid technique by Ni and Li [137]. Here, a mixture of boron, silicon, and iodine powders with the weight ratio 40:1:1 was placed into a smaller quartz boat and placed at one end of a quartz tube of diameter 19.05 mm, while a single crystal Si(100) substrate was placed at the other end of the tube after coating with a 3- or 20-nm Au film (the former was used for crystalline boron nanobelt growth and the latter was used for amorphous boron nanobelt growth). Crystalline boron nanobelts were grown on Si(100) at 1100 °C with the source maintained at 1150 °C [137]. Most of the crystalline nanobelts have a width-to-thickness ratio of 2 and are covered with a layer of amorphous silicon oxide. Amorphous boron nanobelts were grown when the Si substrate temperature was 1050–1070 °C. From these results, the authors pointed out that the temperature gradient in the system is an important factor in addition to the high cooling rate to achieve crystalline boron nanobelts.

#### 3.3.2. Boron nanoribbons

In 1990, Komatsu and Moriyoshi [9] reported that β-rhombohedral boron nanoribbons were synthesized on a Si(100) substrate, as well as whiskers and platelets by plasma-enhanced CVD with 2 kW from a gas mixture of B_2_H_6_ (0.25–1.5 vol%), H_2_ (5–30 vol%), and He at 700–880 °C, where the flexible ribbon-like morphology was observed by SEM, though the precise width-to-thickness ratio was not examined[Bibr CIT0009].

On the other hand, in 2004, single-crystalline α-tetragonal boron nanoribbons of a width of 200–500 nm and thickness of ~20 nm were synthesized at low temperature (630–750 °C) and low pressure (200 mTorr) by pyrolysis of diborane gas in a quartz tube furnace without a catalyst by Xu et al. [138]. The nanoribbons were confirmed by EELS to be composed of boron. SEM images show that the nanoribbons are typically twisted and the edges of ribbons are sometimes not straight, but rather a zigzag shape. By applying the same method, crystalline α-tetragonal boron nanoribbons of thickness 16 nm were reported to be synthesized by Jash and Trenary in 2009 [139].

#### 3.3.3. Borophene nanoribbons on Ag(110)

**Figure 10.**
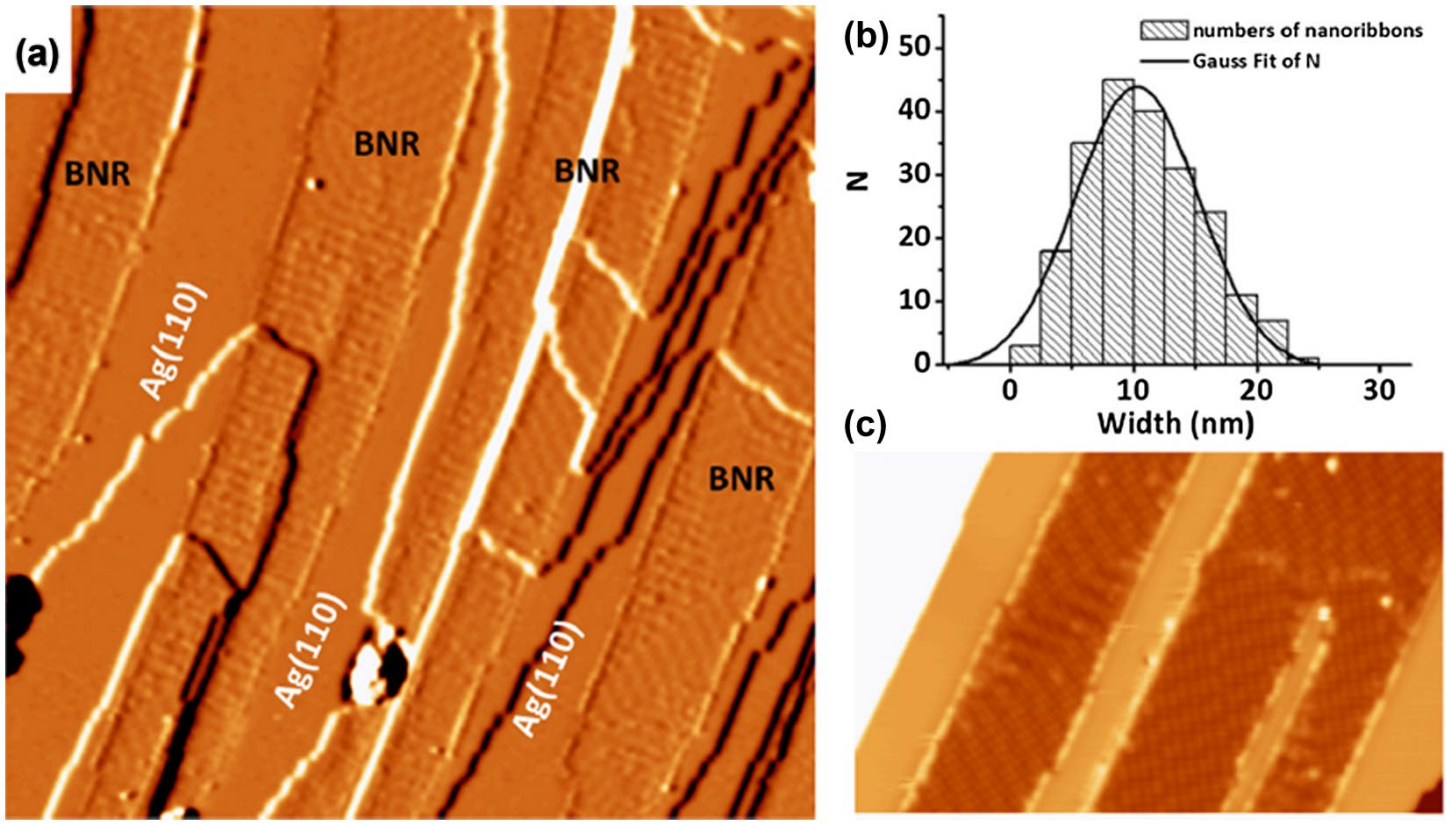
Borophene nanoribbons on Ag(110) surface. (a) A derivative STM image shows boronphene nanoribbons grown on Ag(110). The image size is 100 × 100 nm^2^. The nanoribbons run across the substrate steps without losing continuity. (b) Histogram of nanoribbon width. Gaussian fitting is shown as a black line. (c) High-resolution STM image of two boronphene nanoribbons. Image size: 50 × 30 nm^2^. The bias voltages of STM images are (a) −4.5 V and (b) −1.9 V. Reprinted with permission from Ref. [140]. Copyright 2017 American Physical Society.

**Figure 11.**
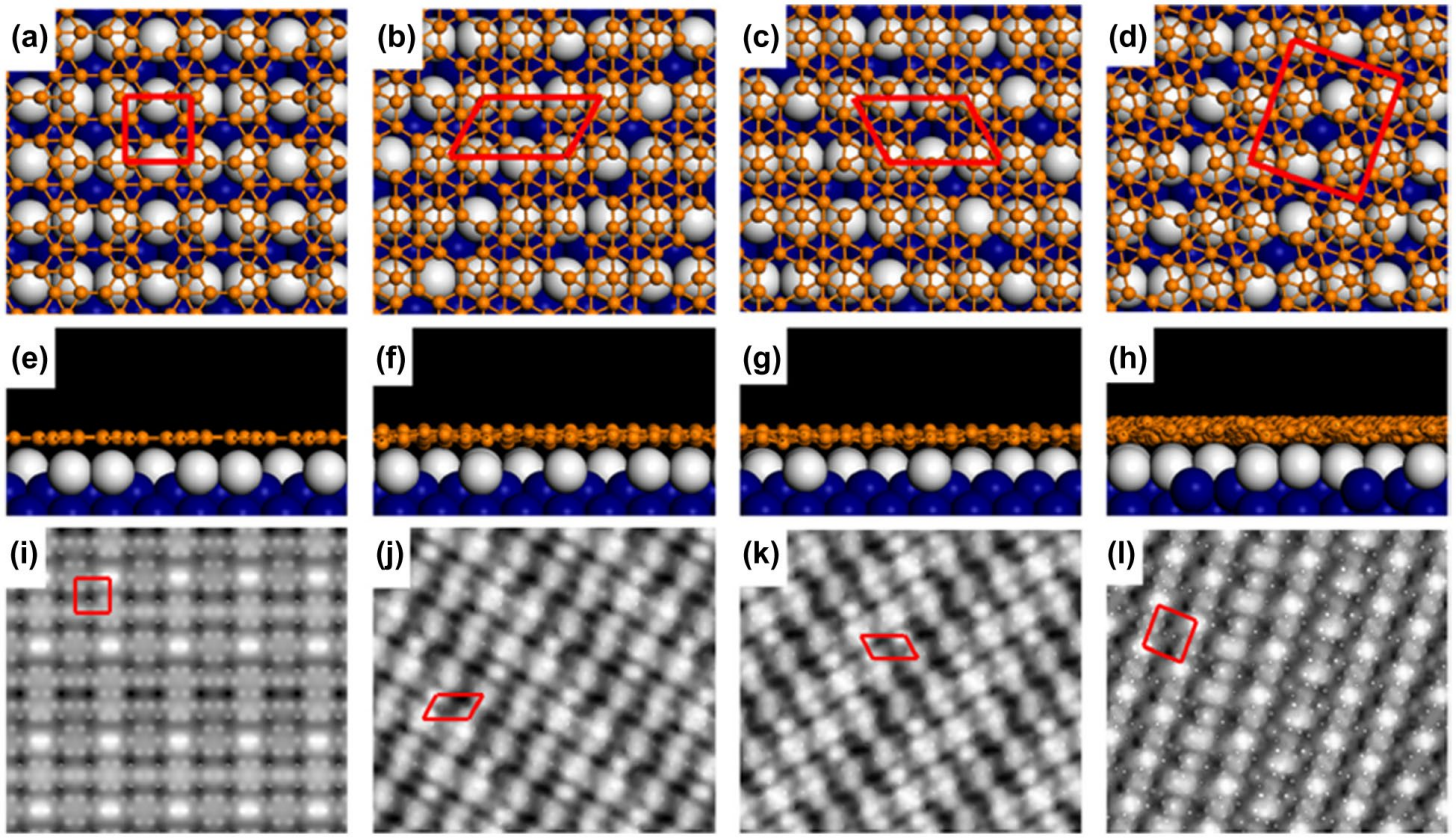
Atomic structures of borophene nanoribbons on Ag(110) optimized by DFT. (a)–(d) Top and (e)–(h) side views of optimized P1–P4 borophene nanoribbons on Ag(110) surface, respectively. Color codes: B, small orange spheres; topmost Ag, large white spheres; lower Ag, large blue spheres. (i)–(l) Simulated STM images for P1–P4 based on the calculated electronic density (0–2 eV above the Fermi level). The red frames correspond to the observed unit cells in STM images. Reprinted with permission from Ref. [140]. Copyright 2017 American Physical Society.

In 2017, Zhong et al. reported synthesis of single-atom-thick borophene nanoribbons by self-assembly of boron on Ag(110) surface [140]. More specifically, boron with 99.9999% purity was evaporated using an electron-beam evaporator on the Ag(110) surface, while the surface was kept at a temperature of 570 K in an ultrahigh vacuum with base pressure of 2 × 10^−11^ Torr. The scanning tunneling microscopy (STM) studies reveal high quality borophene nanoribbons: all the ribbons are along the [−110] direction of Ag(110), and can run across the steps on the surface as shown in Figure 10(a). The width of ribbons is distributed in a narrow range around 10.3 ± 0.2 nm (Figure 10(b)). High resolution STM images revealed four ordered surface structures in borophene nanoribbons (Figure 10(c)). Combined with density functional theory (DFT) calculations, authors found that all four structures of borophene nanoribbons (named P1, P2, P3, and P4) consist of the boron chains with different widths, separated by hexagonal hole arrays (Figure 11). More specifically, the structures of P1, P2, P3, and P4 are assigned as the same structure of so-called χ_3_, β_12_, β, and β_8_ borophene sheet, respectively, predicted by the previous theoretical study [37]. To represent the nanoribbon character, the authors named them BC(2,2), BC(2,3), BC(3,4), and BC(4,4), respectively, where *n* and *m* in BC(*n*, *m*) denote the number of atoms in the widest and narrowest regions of a single boron chain.

## 4. Two-dimensional (2D) boron nanomaterials

2D materials consisting of a single or a few layers of atoms exhibit superior performance compared to conventional materials or their bulk counterparts in a variety of applications, because of their unique properties, including their flexibility, high specific surface area, and quasi-2D electron confinement [141–146]. In the case of boron, small clusters with 2D structures were predicted in the 1990s [13–19] and then synthesized in gas phase after 2013 [84,94,147,148], while large-sized boron nanosheets or borophene were synthesized after 2015 [46,47,81,149–153]. The experimental realization of borophene stimulates the theoretical study and several applications of borophene, such as hydrogen storage, batteries, catalysts, electronics, superconductors, and/or mechanically strong components have been predicted together with novel structural, electronic, thermal, optical, and mechanical properties [20,49–78,80,81].

### 4.1. Boron nanosheets

**Figure 12.**
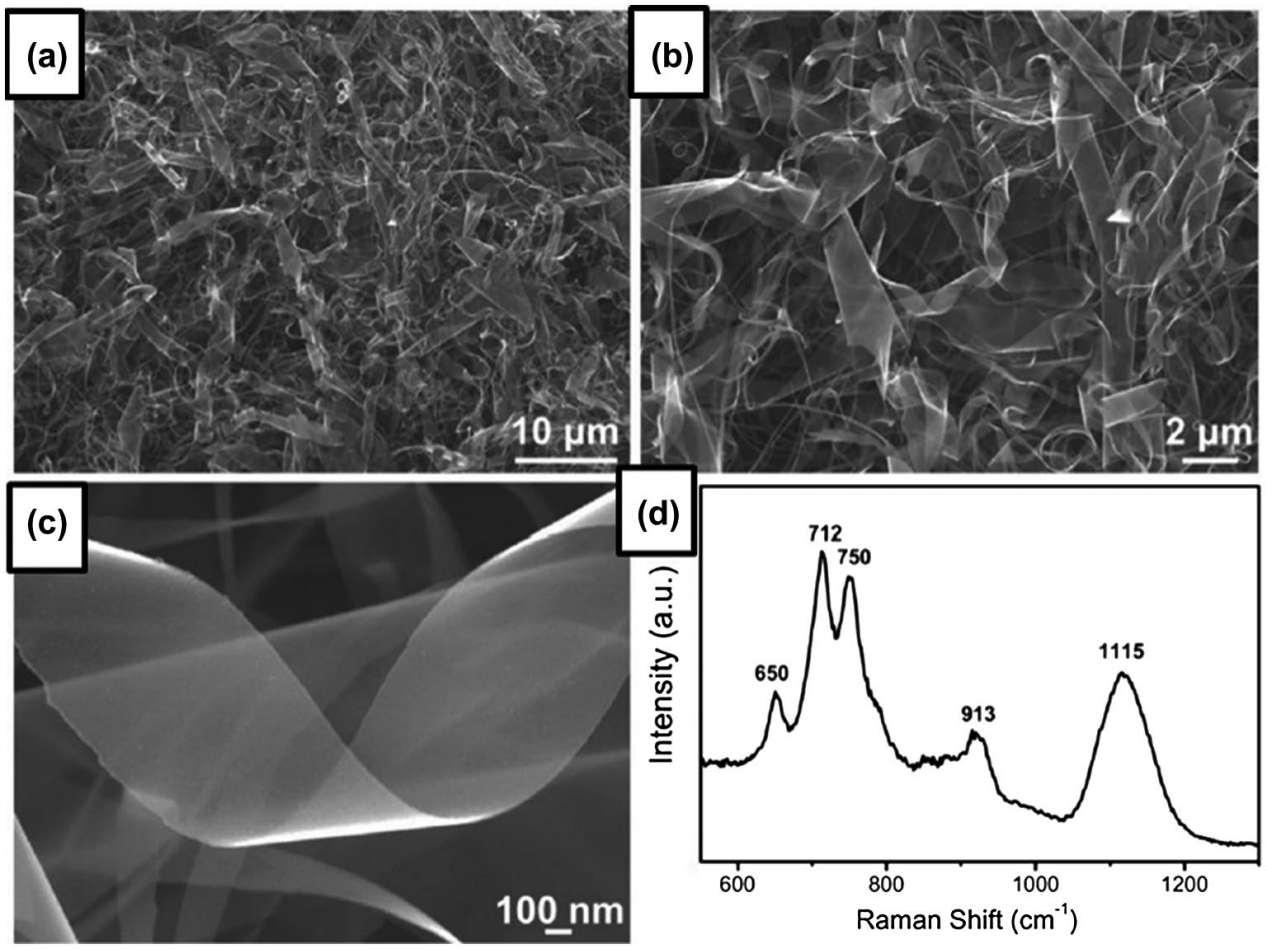
(a)–(c) SEM images for the ultrathin boron nanosheets at different magniﬁcations. (d) Micro-Raman spectrum of the ultrathin boron nanosheets at room temperature. Reprinted from Ref. [81] which is an open access article published by WILEY-VCH Verlag GmbH & Co. KGaA, Weinheim.

Xu et al. reported the synthesis and application of ultrathin single-crystalline boron nanosheets [81]. The nanosheets were synthesized on Si wafers by an effective vapor-solid process via thermal decomposition of diborane without catalyst in a 30-mm external-diameter quartz tube at 950 °C by introducing a mixture gas (5% diborane and 95% argon) at a flow rate of 5 sccm for 120 minutes at 8 Pa of the reaction pressure followed by cooling the sample to room temperature naturally (without quench). Figure 12(a) and (b) show SEM images of the nanosheets, which have a width ranging from tens of nanometers to 3 μm and a length of 3–20 μm. Owing to the large width, these products can be called nanosheets rather than nanoribbons. The nanosheets are almost transparent in the SEM image (Figure 12(c)), indicating the ultrathin thickness. The peaks in the Raman spectrum of the nanosheets shown in Figure 12(d) coincide with those of the α-tetragonal boron phase, which agrees with their SAED pattern analysis. Based on the high-resolution TEM observations, the average thicknesses of the nanosheets are estimated as ≈8–12 nm. EELS revealed that the nanosheets consist of boron and there is no oxygen, carbon, or other impurities. These nanosheets show excellent ﬁeld emission performances with a low turn-on ﬁeld of 3.60 V μm^−1^ and good stability. Moreover, the nanosheets have an intrinsic p-type semiconductor behavior with a carrier mobility of approximately 1.26 × 10^−1^ cm^2^ V^−1^ s^−1^. The photodetector device fabricated from single-crystalline ultrathin boron nanosheets further demonstrates good sensitivity, reliable stability, and fast response, obviously superior to other reported boron nanomaterials [81]. The reports by Xu et al. thus show the great potential of boron nanosheets as a material for applications in field emitters, interconnects, integrated circuits, and optoelectronic devices.

### 4.2. Borophene on Ag(111)

A single-atomic two-dimensional layer of boron named borophene has been reported to be grown by the physical evaporation of boron atoms on Ag(111) in ultra-high vacuum by parallel works of Mannix et al. [46] and Feng et al. [47]. They have observed different phases of the borophene sheets on Ag(111). Subsequently, additional new two phases of borophene on Ag(111) are reported by Zhong et al.[153]. All of the borophene sheets on Ag(111) are reported to be metallic.

The fact that only a few structures were found on Ag(111) surface is rather surprising since freestanding borophene were predicted to be polymorphic with a tremendous number of boron structures sharing competing binding energy near the global minimum [37]. It may indicate that the specific interfacial interactions between the substrate and borophene play important roles in determining the morphology of borophene as noted by Zhong et al. [140]. Here, the reported phases of borophene on Ag(111), namely striped, homogeneous, S1, S2, S3, and S4 phases are described.

#### 4.2.1. Striped and homogeneous phases

In 2015, Mannix et al. [46] reported two types of monolayer crystalline boron sheets (borophene) synthesized by physical vapor deposition of boron on Ag(111) at 550 °C under ultrahigh vacuum[Bibr CIT0046]. The phases are identified as the striped and homogeneous phases. The striped phase consists of regions with prominent stripe features that are composed by the rectangular lattice. The homogeneous phase is composed of chain-like atomic-scale features that are buckled vertically out-of-phase with respect to their neighbors. The relative concentration of the phases depends upon the deposition rate. Low deposition rates favored the striped phase and resulted in the growth of striped-phase nanoribbons on Ag(111). At higher deposition rates such as 0.1 monolayer/min more of the homogeneous islands are observed by STM. Increasing growth temperatures favored the striped phase, while the homogeneous phase could be observed when boron atoms were deposited at a lower temperature such as 450 °C. These results suggest that the homogeneous phase is metastable relative to the striped phase.

**Figure 13.**
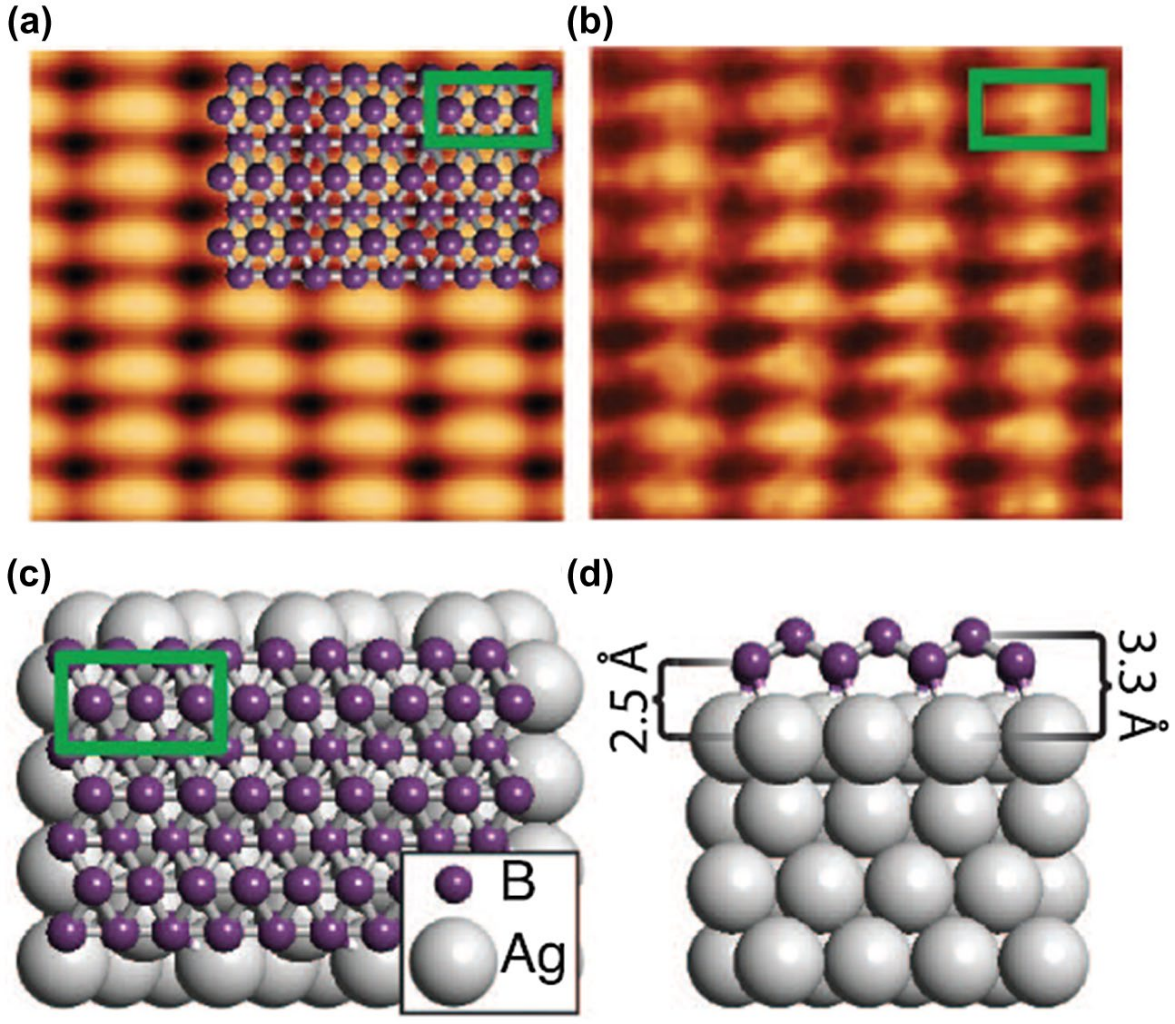
(a) Simulated empty states STM image (*V*_sample_=1.0 V), with overlaid atomic structure and unit cell of 0.500 nm by 0.289 nm. (b) Experimental STM image (*V*_sample_ = 0.1 V, *I*_t_=1.0 nA) of borophene was dominantly observed when it was grown at a high temperature of 700 °C, with overlaid unit cell of 0.51 nm by 0.29 nm. Top (c) and side (d) views of the low-energy monolayer structure corresponding to the δ_6_-type borophene sheet (unit cell indicated by the green box). From [46]. Reprinted with permission from AAAS.

An STM image of the striped phase is shown in Figure 13(a). The observed STM image is well consistent with the simulated STM image (Figure 13(b)) as shown in Figure 13(c) and (d). Auger electron spectroscopy and X-ray photoelectron spectroscopy show that these sheets are composed of boron atoms. This striped phase (borophene structure shown in Figure 13) corresponds to that of δ_6_-type borophene sheet theoretically predicted earlier [37]. Based on the scanning tunneling spectroscopy (STS) results with gapless local density of states, the authors concluded that the striped phase has a metallic nature. The other metastable phase, homogeneous phase, was proposed to arise from regular perturbations of the buckled triangular lattice, or a similar structure, occurring due to substrate interactions and phonon instabilities [46,154].

**Figure 14.**
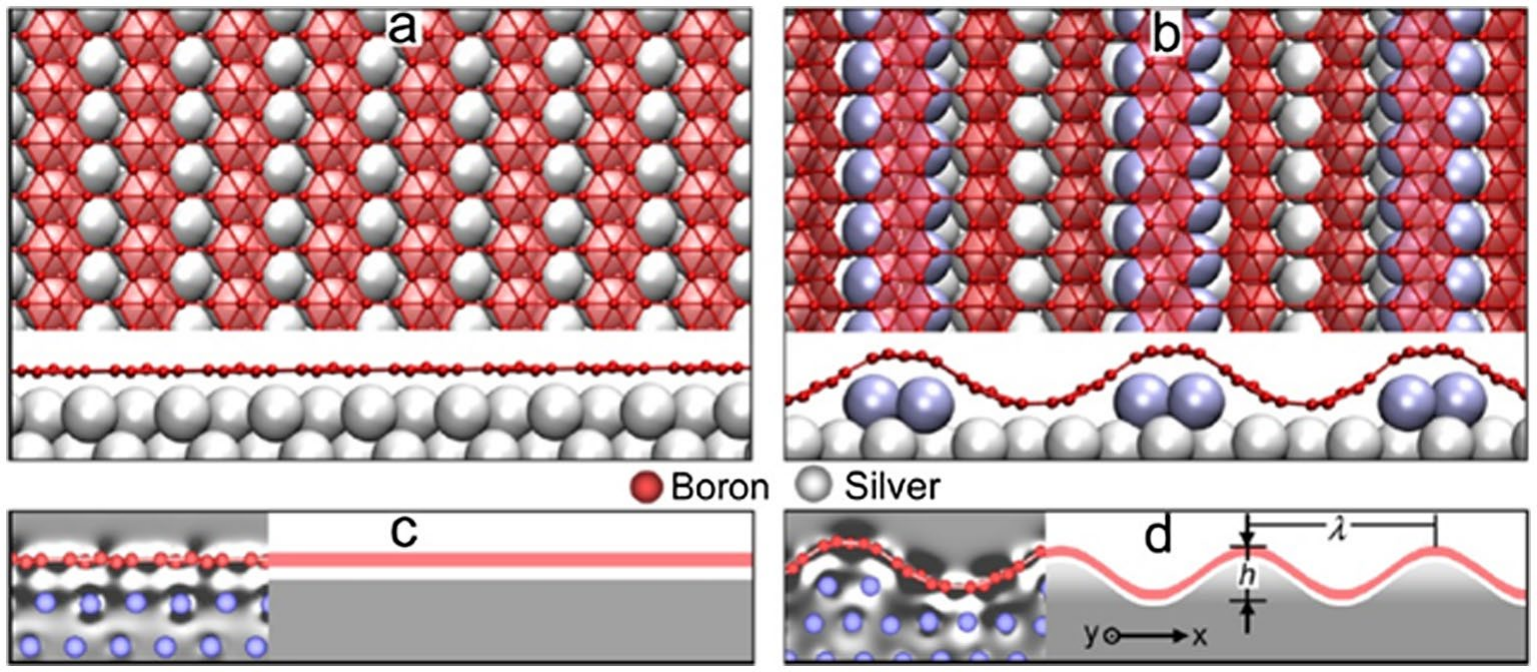
Atomic geometry of periodically undulated borophene on Ag(111). (a) Front (top) and side (bottom) views of a β_12_-type borophene sheet on silver. (b) Front (top) and side (bottom) views of an undulated β_12_-type borophene sheet on reconstructed Ag(111). The topmost Ag atoms are colored blue for clarity. (c), (d) Schematic continuum models for the (c) planar and (b) undulated β_12_-type borophene sheets on a compliant substrate. Insets illustrate slices of charge redistribution between the B sheet (red) and Ag (blue), where dark and light colors represent charge depletion and accumulation (0.001 e/Å^3^) regions, respectively [152]. Copyright 2016 American Chemical Society.

In a recent study, the striped phase observed by STM was explained by the undulated so-called β_12_-type borophene sheet (the structure of which was predicted in an earlier theoretical work [37]) on a reconstructed Ag(111) surface [152] as shown in Figure 14. Here, based on DFT calculations the authors show that the bending stiffness of the β_12_-type borophene sheet is as small as 0.39 eV along the hollow hexagon, which is about one-fourth of the 1.5 eV for graphene, indicating the greater flexible nature of borophene compared to graphene.

In a more recent study, the self-assembly of lateral heterostructures between homogeneous phase borophene and perylene-3,4,9,10-tetracarboxylic dianhydride (PTCDA) on Ag(111) was reported [155]. They first deposited boron on a Ag(111) thin film (~300 nm thick) on a mica substrate in UHV by electron beam evaporation of a pure boron rod. By maintaining the substrate at a temperature of ~480 °C, pure homogeneous-phase borophene is formed. Subsequently, PTCDA molecules were deposited by thermally evaporating PTCDA from an alumina-coated crucible. Fine-tuning of the evaporation temperature and duration allows precise, layer-by-layer growth of self-assembled PTCDA on Ag(111). The preferential assembly of PTCDA on Ag(111) compared to borophene leads to the spontaneous formation of borophene/PTCDA lateral heterostructures. The preferential assembly of PTCDA was ascribed to the higher adsorption enthalpy of PTCDA on Ag(111) and lateral hydrogen bonding among PTCDA molecules, which was confirmed by the analysis with molecular dynamics simulations. The realization of a borophene-based heterostructure will inform emerging efforts to integrate borophene into nanoelectronic applications.

#### 4.2.2. S1 and S2 phases

**Figure 15.**
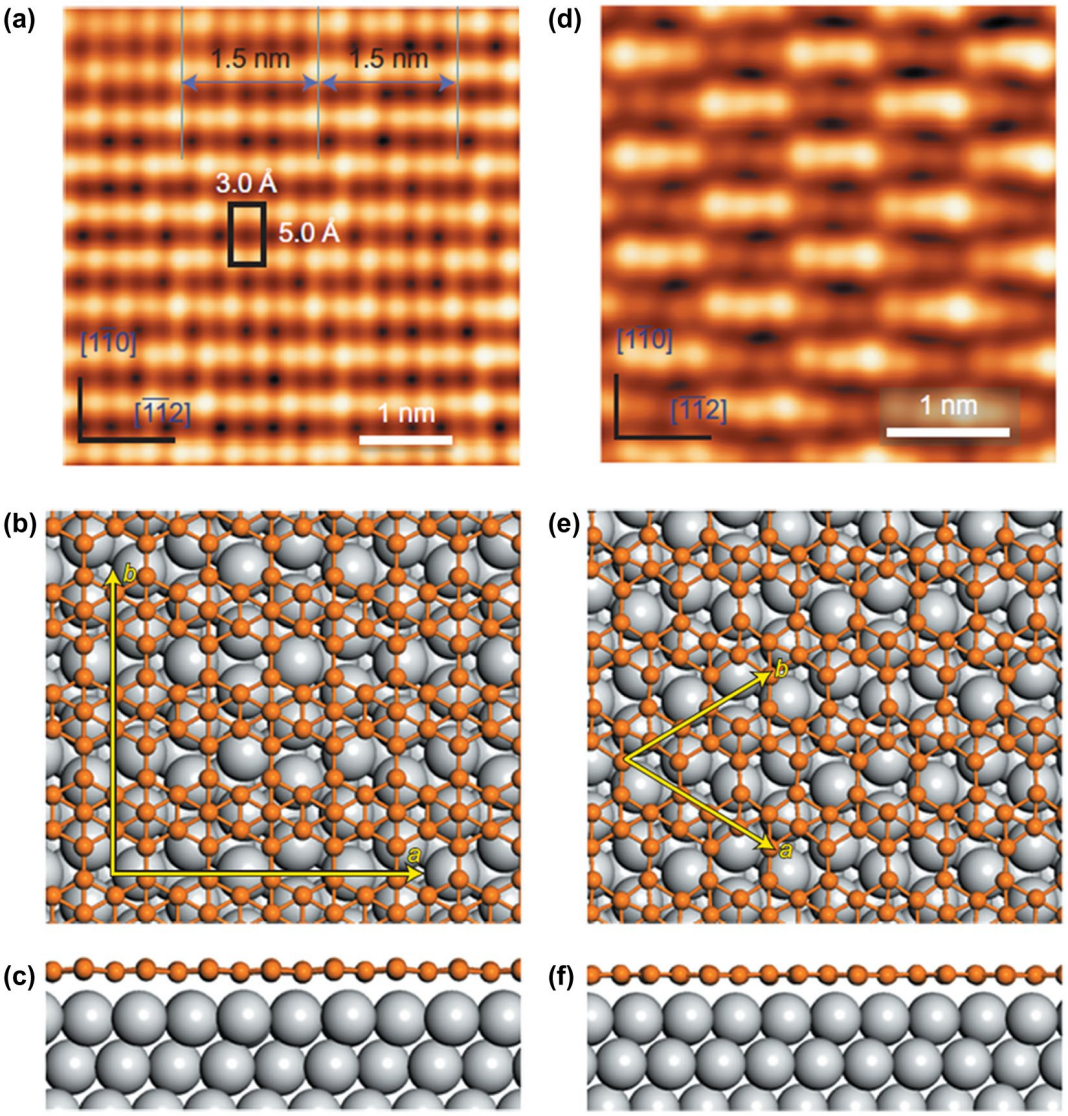
(a) STM image of β_12_-type borophene sheet on Ag(111) (S1 phase) grown on 570 K. Unit cell of 0.50 nm × 0.30 nm is marked by a black rectangle. Top (b) and side (c) views of β_12_-type borophene sheet on Ag(111). (d) STM image of χ_3_-type borophene sheet on Ag(111); this phase (S2 phase) appeared only after annealing at 650 K. Top (e) and side (f) views of χ_3_-type borophene sheet on Ag(111). Reprinted with permission from Macmillan Publishers Ltd from Ref. [47]. Copyright 2016.

Independent of Mannix et al., in 2016 Feng et al. [47] reported two types of borophene sheets grown on Ag(111)[Bibr CIT0047]. Borophene with a different structure from striped phase was grown when the substrate Ag(111) temperature was 300 °C (a much lower temperature compared with 550 °C for Mannix et al. [46]). Figure 15(a) is the corresponding STM image of borophene on Ag(111). This borophene was named as S1 phase and assigned as β_12_-type borophene sheet on Ag(111) without reconstruction (Figure 15(b) and (c)). The growth of β_12_-type borophene sheet on Ag(111) is consistent with a previous prediction of the borophene structure on Ag [156]. They also found that the β_12_-type borophene sheet transformed to the different type of sheet named the S2 phase by annealing the sample to ~380 °C. An STM image of the S2 phase is shown in Figure 15(d). Mannix et al. noted that they have observed this S2 phase previously as the ‘homogeneous phase’ [46,154]. Feng et al. assigned the S2 phase as χ_3_-type borophene sheet [37] on Ag(111), the structure of which is schematically shown in Figure. 15(e) and (f). Both β_12_-type and χ_3_-type borophene sheets show a planar structure on Ag without corrugation. At higher boron coverage, the growth of 3D boron clusters is observed instead of the growth of multilayer borophene sheets, suggesting that the growth of monolayer boron sheets is assisted by the interaction with the substrate Ag(111). Later angle-resolved photoelectron spectroscopy measurements revealed that β_12_-type borophene sheets on Ag(111) (S1 phase) have a metallic nature [157] and exhibit Dirac fermions [79].

#### 4.2.3. S3 and S4 phases

**Figure 16.**
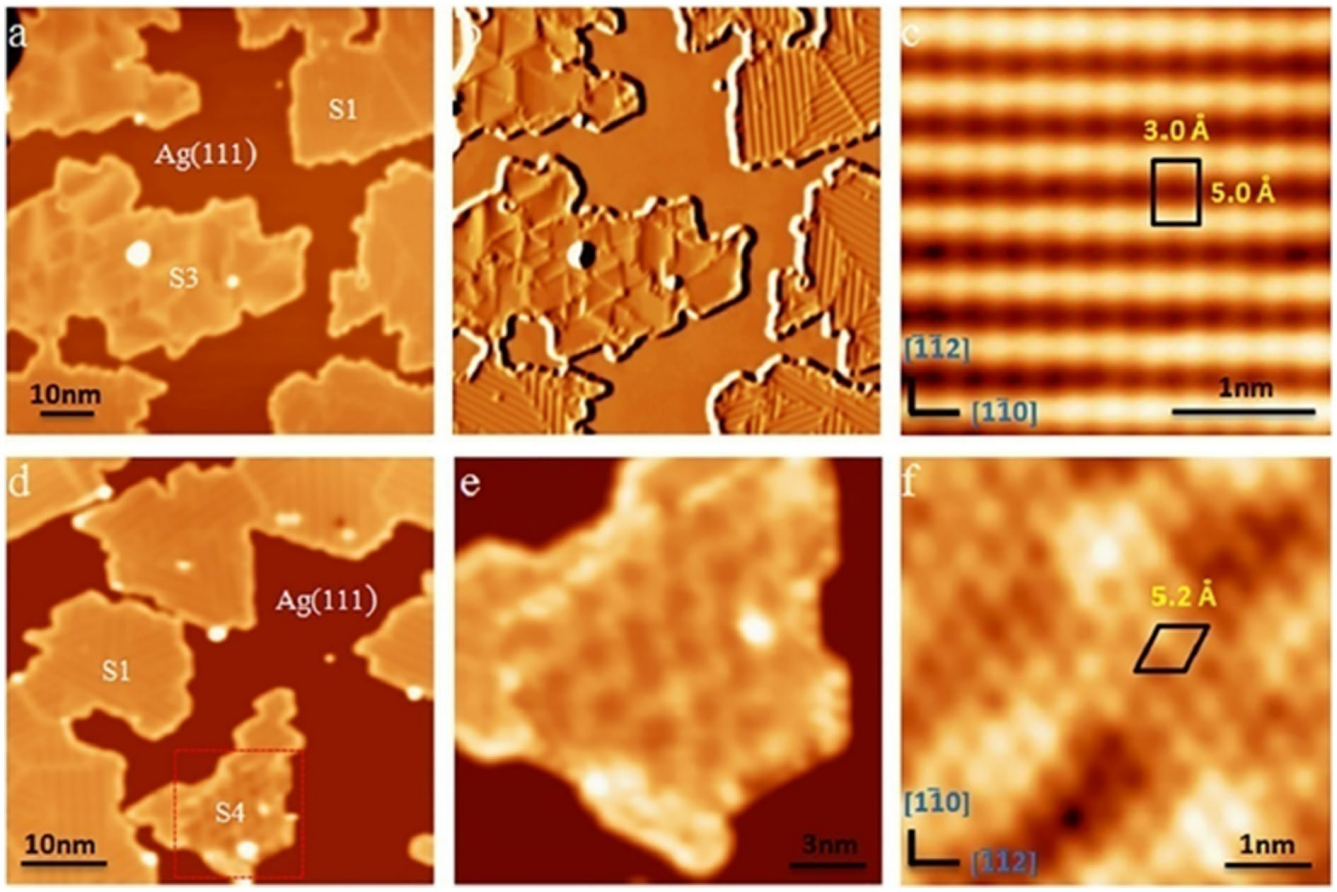
STM images of two metastable 2D boron sheets on Ag(111). (a) STM topographic image of boron structures on Ag(111). The boron islands are labelled as ‘S1’ and ‘S3’ phases. (b) The derivative STM image of (a). (c) High-resolution STM image of the S3 phases. The S3 unit cell is marked by a black rectangle. (d) STM topographic image of boron structures on Ag(111). The boron islands are labelled as ‘S1’ and ‘S4’ phases. Most boron islands shown in the image are S1 phase. (e) STM image obtained on the area marked by the red dotted rectangle in (d). (f) High-resolution STM image of the S4 phase. The S4 unit cell is marked by a black rhombus [153]. Copyright 2017 IOP Publishing.

**Figure 17.**
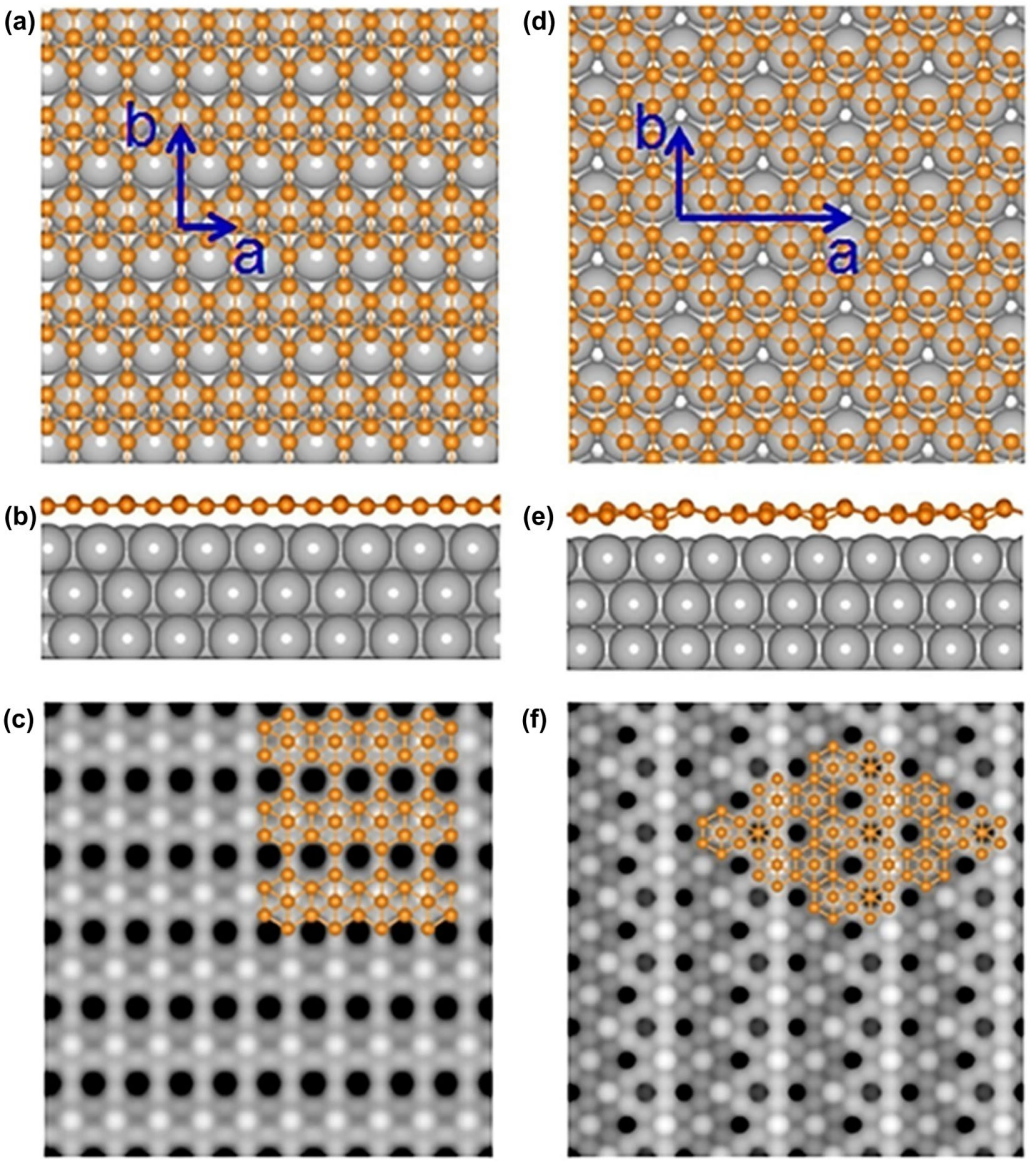
Structure models of the S3 and S4 phases of boron sheets based on DFT calculations. (a) and (b) Top and side views of the S3 model, which correspond to the β_12_ sheet of 2D boron on a Ag(1 1 1) surface. (c) Simulated STM topographic image of the β_12_ sheet. (d) and (e) Top and side views of the S4 model, which correspond to the α sheet of 2D boron on Ag(1 1 1). The α sheet was buckled by 1.1 Å in the z axis when put on Ag(1 1 1). (f) Simulated STM topographic image of the α sheet. The orange and grey balls in (a), (b), (d), and (e) represent boron and silver atoms, respectively. The basic vectors of the super cell, including the Ag(1 1 1) substrate, are marked by blue arrows. Models of the β_12_ and α sheets are superimposed on their simulated STM images [153]. Copyright 2017 IOP Publishing.

In a 2017 report by Zhong et al. [153], two additional reproducible new phases (named the S3 and S4 phases) have been found for borophene on Ag(111). Both of these phases are confirmed to be metallic based on STS measurements. They deposited boron atoms on Ag(111) at 570 K, which is in a temperature window where the S1 phase formed as described above [47]. Figure 16(a) shows an STM image of the surface after the boron deposition. The S1 phase (β_12_-type borophene sheet on Ag(111)) is indeed dominantly formed as much as 92%, while the other phase of 8% is one of the new phases named S3. The S3 phase shares the identical atomic structure of the S1 phase but has a different rotational relationship with the substrate, and thus exhibits very different features in STM images as shown by the derivative STM image in Figure 16(b). More specifically, the short side of the unit cell of the S3 phase is in the [1 -1 0] direction of Ag(111) (Figure 16(c)), while that of the S1 phase is in the [-1 -1 2] direction of Ag(111). As shown in Figure 16(a) and (b), no moiré patterns were then observed for S3 phase, while 1.5 nm-wide parallel stripes were observed for S1 phase which come from the commensuration between β_12_ structure and the lattice of the Ag(111) substrate in the [-1 -1 2] direction. Interestingly, the lower population phase of S3 has a perfect lattice matching between borophene and Ag(111) compared with that for the higher population phase of S1. As shown in Figure 17(a)–(c), the triangular lattice *a* and *b* for the S3 phase are 5.0 Å and 3.0 Å for borophene, while the corresponding distances for Ag(111) are 5.02 Å and 2.9 Å. Thus, no moiré patterns were observed for the S3 phase. The lower population of the S3 phase was ascribed to the slightly smaller total formation energy for S3 (6.24 eV per atom) compared to that of S1 (6.32 eV per atom) based on the DFT calculations [47, 153].

The other new phase of S4 is more rarely observed compared to the S3 phase on the Ag(111) surface. As shown in Figure 16(d) of another area of the same sample, most islands in this image are S1 islands, while there is a small island exhibiting a different structure, as denoted by the S4 phase. Figures 16(e) and (f) are magnified STM images of the S4 phase where only the inner part of the island in Figure 16(e) exhibits a different structure, while the two edges of the island are still the S1 structure. As shown in the high resolution STM image of this phase (Figure 16(f)), it has a hexagonal crystal lattice in addition to the global moiré pattern. The rhombic unit cell, as marked in Figure 16(f) has a side length of 5.2 Å, which is very close to that of the so-called α-sheet (5.0 Å), which is the structure theoretically predicted [13–45]. Based on the comparison with a simulated STM image using DFT, the S4 phase was then assigned as the α-sheet on Ag(111) with buckles by 1.1 Å in the z-direction as shown in Figure 17(d)–(f). According to the atomic structure of boron sheets, the density of the hexagonal holes in S1 (1/6) is larger than S4 (1/9), which means that the β^12^ sheet (S1) is slightly electron positive relative to the α-sheet (S4). From the view of electric neutrality, the fact that the S4 resides with the S1 phase may help in stabilizing the electron surplus in S4 on the Ag(111) [47, 153].

### 4.3. Borophene on Cu foil

**Figure 18.**
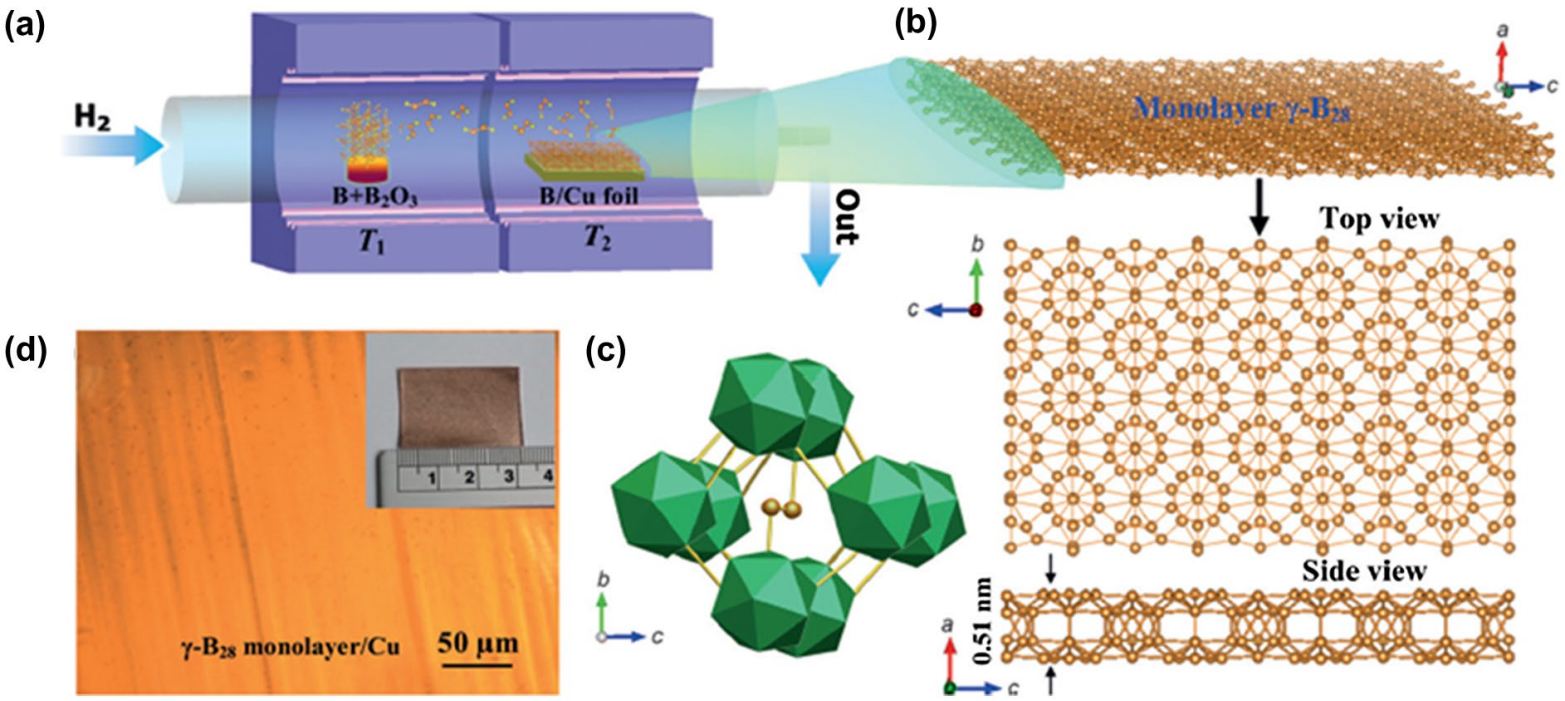
(a) Schematic representation of the two-zone furnace used to obtain atomically thin γ-B_28_ films by CVD. The temperatures of the source zone (*T*_1_) and substrate zone (*T*_2_) were set as 1100 °C and 1000 °C, respectively for the synthesis. (b) Top and side views of the monolayer. (c) Polyhedral structure of the basic unit cell of the monolayer is shown in the bc projection. Boron atoms forming dumbbells are shown as orange spheres. (d) Optical image of a monolayer on Cu foil. Inset: photograph of the monolayer on Cu foil. Reproduced from Ref. [150] by permission of John Wiley & Sons Ltd.

Tai et al. [150] reported atomically thin crystalline boron films synthesized on copper foils at 1000 °C for 1 hour by CVD using a mixture of pure boron and boron oxide powders at 1100 °C as the boron source and hydrogen gas as the carrier gas (Figure 18(a))[Bibr CIT0150]. The structure was identified as 2D γ-B_28_, which is composed of icosahedral B_12_ units and B_2_ dumbbells, as shown in Figure 18(b). It features orthorhombic γ-B_28_ cells with a unit cell of 28 atoms (*a* = 5.054 Å, *b*=5.620 Å, *c*=6.987 Å) and a space group *Pnnm*, and it can be regarded as a boron boride (B_2_)^δ+^(B_12_)^δ-^ because of the charge transfer between these two components [158]. The basic unit cell for *b*-*c* projection is shown in Figure 18(c). The structure is built of B_12_ icosahedra linked into a 3D network by B_2_ dumbbells: the dumbbells are aligned almost parallel to the *a* axis, and nearly rectangular channels along the axis are filled with boron chains. A boron thin film was controllably prepared over the entire surface of a Cu foil with a size of 2 × 3 cm^2^ (Figure 18(d)). The authors transferred it onto a 285-nm thick SiO_2_/Si substrate by removing the Cu foil using dilute ferric chloride (FeCl_3_) solution. Atomic force microscopy analysis of the transferred boron sheet on SiO_2_/Si shows that the boron sheet consists of a monolayer. Thus, the γ-B_28_ monolayer sheet can also be called borophene (γ-B_28_ type borophene). Interestingly, in the case of γ-B_28_ type borophene, the building block of B_12_ icosahedra is still presented, contrary to the cases of β_12_-type, χ_3_-type, α-type and δ_6_-type borophene sheets.

## 5. Perspective and future directions of boron nanomaterials

A significant number of theoretical predictions have been reported about the advantages of boron nanomaterials for applications such as hydrogen storage, batteries, catalysts, electronics, superconductors, and/or mechanically strong components [20,49–78,80,81]. Compared to the theoretical predictions, experimental studies on the application of boron nanomaterials are limited. However, as described above, the individual boron nanotube shows excellent field emission properties, with a high stable current of approximately 80 μA and a current density of 2 × 10^11^A/m^2^ which are very close to those of carbon nanotubes [131]. The excellent field emission properties of boron nanowires, such as low turn-on field and high current endurance, have also been reported [123–129]. Uniform crystalline boron nanowires with square (25 μm × 60 μm) patterns have been grown over a large area by CVD on a Si substrate and the patterns show bright and uniform performance of field emission [129]. These results suggest that the boron nanotubes and nanowires are a promising material for application as field emission displays. The boron nanowires also show a very high specific fracture strength of 3.9 GPa·cm^3^/g and specific elastic modulus of 130.6 GPa·cm^3^/g, which are one to two orders of magnitude larger compared to many reported nanostructures [122]. These results suggest that the boron nanowires are a promising material for application as lightweight reinforcing fillers. The photodetector device fabricated from single-crystalline ultrathin boron nanosheets demonstrates good sensitivity, reliable stability, and fast response [81], thus showing the great potential of the boron nanosheets as a material for applications in field emitters, interconnects, integrated circuits, and optoelectronic devices.

Here, the concept of the building block for creating new materials is described together with the future directions of synthesis, functionalization, and mass production of boron nanomaterials rather than merely introducing theoretical predictions for the applications.

### 5.1. Boron nanomaterials as building blocks

As described above, boron consists of the building blocks of B_12_ icosahedra or icosahedral fragments, but nanomaterials of boron (at least in the case of borophene except for the γ-B_28_ type) are not composed by them, owing to their low dimensionality. Here, it should be noted that a wide variety of boron nanomaterials themselves could be building blocks from the viewpoint of large-scale materials. Indeed, combining 2D materials through layer stacking in a controlled manner has already been focused on and is reported to produce several novel functionalities in the form of new 3D layered materials (van der Waals heterostructures) [159]. Therefore, the synthesis of new boron nanomaterials opens several pathways for the applied use of new materials. That is, boron nanowires, nanoribbons, nanobelts, nanotubes, and nanosheets are all building blocks and we can design and create materials with new functionalities and properties by combining these boron nanomaterials with other existing nanomaterials, molecules, atoms, and/or ions.

### 5.2. Transfer of borophene sheets for the application

In the case of γ-B_28_ type borophene grown on Cu, it was transferred to a 285-nm thick SiO_2_/Si substrate by removing the Cu foil using dilute ferric chloride (FeCl_3_) solution [150]. On the other hand, each type of borophene sheet on Ag(111) is currently just a superstructure of an adatom layer deposited on the single crystal surface from the viewpoint of surface science, because they are connecting with the substrate used for growth. It is thus uncertain whether we can obtain freestanding borophene sheets while keeping the structures formed on Ag(111) because the borophene sheets grown on Ag(111) consist of the boron bonding configuration without the typical building blocks of B_12_ icosahedra.

The presence of a substrate is not a problem for electronic applications, provided the substrate is compatible with the final device. However, Ag(111) used for the growth of borophene is not suitable for some applications, such as a field-effect transistor. Thus, we need to establish a transfer method of borophene from the substrate used for the growth, Ag(111), to the substrate desired for the application, such as an insulator. One promising transfer method has already been demonstrated in the case of single atomic layers of silicon (silicenes) grown on Ag(111) layers on mica, where capping the surface of silicene on Ag(111) *in situ* by Al_2_O_3_ enables encapsulated delamination transfer of silicene on SiO_2_, and native contact electrode formation can be achieved to realize back-gated silicene transistors [160]. In this case, the sheets are always connected with other elements and thus the stability originating from the interaction with the substrate can be preserved during the process. We can also apply this method to create van der Waals heterostructures or other types of new 3D materials by using borophene as one of the building blocks.

It should be noted that physical vapor deposition on Ag(111) is definitely one of the most useful techniques for growing single-layer sheets composed by single or multiple element(s), as demonstrated by the synthesis of silicene and borophene. Further, new types of single-layer sheet materials will possibly be realized using this method.

### 5.3. Mass production of boron nanomaterials by top-down method

All of the synthesis methods of boron nanomaterials introduced above required a high temperature of the boron source and/or substrate. This is one of the common characteristics of the bottom-up synthesis processes of boron nanomaterials. In the case of 2D materials, on the other hand, liquid exfoliation of 3D layered materials is widely used as a mass production method to obtain free-standing sheets, owing to its low cost and simplicity, which can be classified as a top-down approach compared to syntheses involving material buildup (bottom-up approaches). Sheets are obtained in solution by exfoliation from 3D layered materials through sonication in a surfactant solution, ion/polymer intercalation, or functionalization followed by exfoliation in solvent or suspension [161–164]. To date, numerous 2D materials, including graphene, hexagonal boron nitride (h-BN), transition metal dichalcogenides (e.g. WS_2_ and MoSe_2_), metal halides (e.g. MoCl_2_ and PbCl_4_), and oxides (e.g. MnO_2_ and LaNb_2_O_7_), have been produced by exfoliation in liquid. As the parent material in the top-down synthesis of borophene or borophene-related 2D sheets, we have focused on MgB_2_, a binary compound composed of hexagonal boron sheets alternating with Mg cations. Since MgB_2_ inherently contains 2D boron sheets, it is of interest to determine whether borophene could be formed by simple exfoliation and deintercalation of Mg. According to a recent report by Das et al., however, ultrasonication of water with MgB_2_ at room temperature produces Mg-deficient hydroxyl-functionalized boron nanosheets rather than pure boron sheets [165]. The presence of Mg and hydroxyl species in nanosheets can be explained by the instability of charged boron sheets in water derived from MgB_2_ by exfoliation. In our previous study, we clarified that MgB_2_ is exfoliated in water not by simple Mg deintercalation, but by cation-exchange reactions between protons and Mg cations in MgB_2_, where the produced hydrogen boride sheets subsequently react with water to form Mg-deficient hydroxyl-functionalized boron nanosheets as a result of hydrolysis [166]. We thus consider that the designed ion-exchange method between Mg cations and other cations is a way to produce well-defined, borophene-related, and stable freestanding 2D materials through a top-down approach. In our most recent work, we report the experimental realization of 2D hydrogen boride (HB) sheets with an empirical formula of H_1_B_1_ created by the complete cation-exchange between protons and the magnesium cations in magnesium diboride (MgB_2_), where the ion-exchange was conducted in the acetonitrile or methanol without using water at room temperature [167]. The obtained hydrogen boride sheets can be considered as a new template material for the synthesis of boron nanomaterials. Moreover, the obtained hydrogen boride sheets themselves are very fascinating materials, since theoretical studies predicted that boron hydride sheets would have interesting electronic and mechanical properties [168] as well as high hydrogen storage capacity [169]. Hydrogen boride sheets can also be considered as one of the building blocks of the combining materials, such as van der Waals hetero-structures described above. The method of synthesizing the hydrogen boride sheets can also be applied to synthesize other types of borophene-related nanosheets.

## Disclosure statement

No potential conflict of interest was reported by the author.

## Funding

Takahiro Kondo was supported by the PRESTO program of the Japan Science and Technology Agency (JST) [JSPS KAKENHI grant numbers JP16H00895 and JP16H03823] and MEXT Element Strategy Initiative to Form Core Research Center.
